# Natural scene segmentation dynamics reveal iterative Bayesian inference

**DOI:** 10.64898/2026.01.30.702842

**Published:** 2026-02-02

**Authors:** Tridib K. Biswas, Jonathan Vacher, Sophie Molholm, Pascal Mamassian, Ruben Coen-Cagli

**Affiliations:** 1 Albert Einstein College of Medicine, Dept. of Neuroscience; 2 Université Paris Cité, CNRS, MAP5; 3 Albert Einstein College of Medicine, Dept. of Pediatrics; 4 École Normale Supérieure Paris; 5 Albert Einstein College of Medicine, Dept. of Systems & Computational Biology; 6 Albert Einstein College of Medicine, Dept. of Ophthalmology

**Keywords:** segmentation, psychophysics, Bayesian theories of vision, natural images, perceptual decision-making

## Abstract

The visual system operates by segmenting visual inputs into distinct perceptual objects. Segmentation is dynamic, as revealed by the tempo of perceptual choices and neural activity in visual cortex. Dynamics for natural stimuli however, are poorly understood because natural scene segmentation is ambiguous and subjective. We measured subjective human segmentation maps for natural images using an innovative paradigm, and uncovered richer spatiotemporal dynamics than predicted by current theories of segmentation. To explain these dynamics, we introduced Iterative Bayesian Inference algorithms for segmentation that iteratively integrate visual inputs with the prior expectation that objects are spatially compact. When visual inputs were consistent with such a spatial prior, iterative inference was faster. This predicted relationship between spatial prior and inferential dynamics was evident in our data, and correctly reflected each individual participant’s spatial biases. We conclude that iterative Bayesian inference sets the tempo for a fundamental function of natural vision.

## Introduction

Segmentation is the task of grouping visual inputs into the objects and parts that compose a scene, and separating those objects from each other^1^. Performing segmentation supports varied functions of human vision: from reading these words, to navigating the environment, to understanding the relations between objects. In addition, segmentation influences basic processes in visual perception and may be disrupted in patients with neurologic or psychiatric disorders^2^. Therefore, identifying the computational principles of human segmentation is crucial for understanding human visual perception and its neural substrates.

Although segmentation feels effortless and instantaneous in everyday vision, experiments have found signature dynamics in classical perceptual grouping tasks such as reporting whether two locations cued on a display belonged to the same curve^3–7^, the same letter^8^, or the same object^9–11^. In those tasks, reaction times vary substantially across the visual field, and the larger the distance between two parts of an image, the more time it takes to make perceptual grouping decisions^3–7,9–11^. An influential theory of segmentation posits that this correlation between reaction time and distance arises from how visual attention modulates the activity of retinotopically-organized neurons in the visual cortex. Namely, when attention is directed to a point in the visual field, neurons near the attended–to object in the retinotopic map should become progressively more active^6,12^ due to the sequential spreading of attention across those neurons. This theory, and related computational models^6,11,13–15^, are further motivated by recurrent neural dynamics in early visual cortex^16–18^.

**Figure 1:**
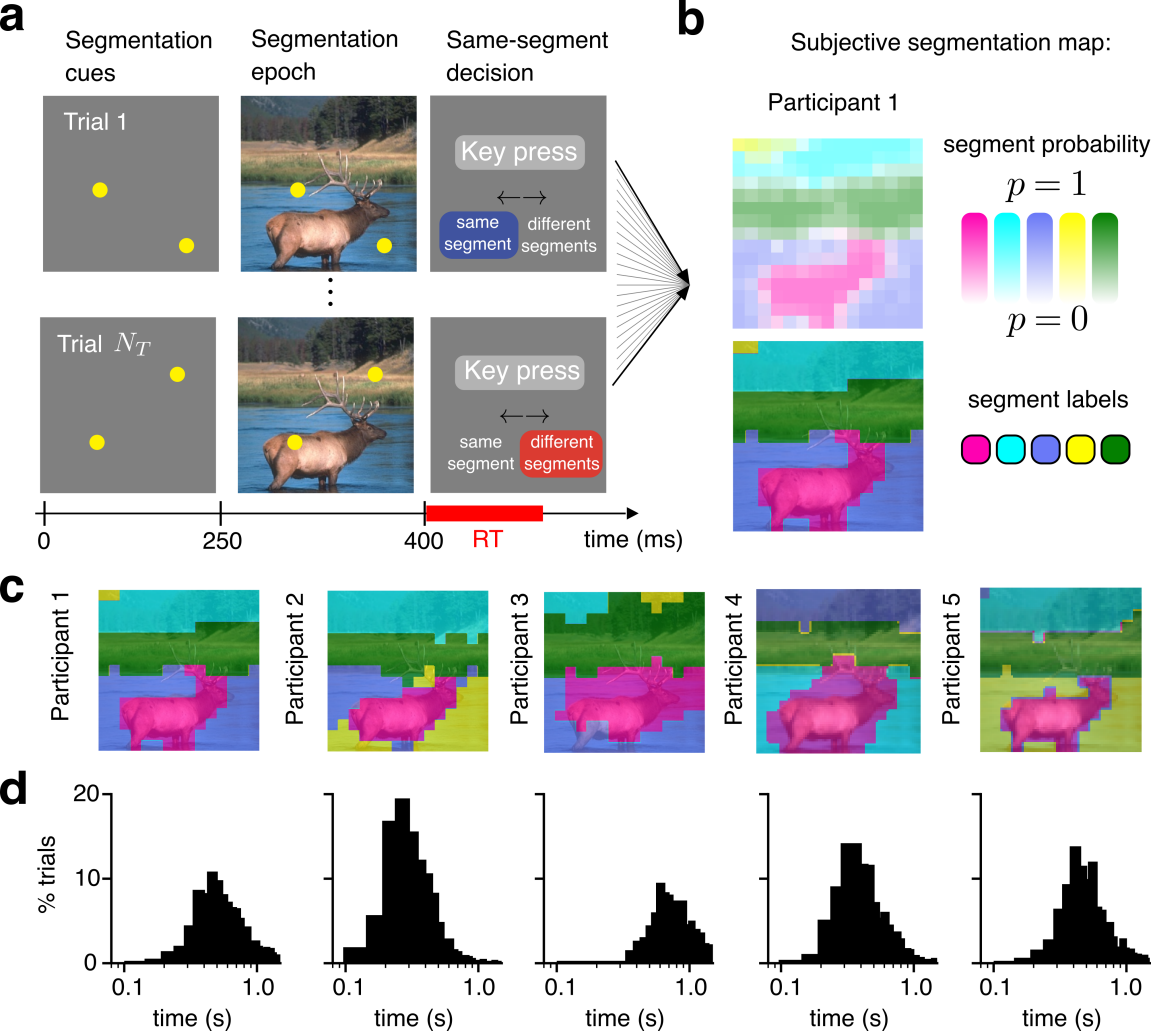
Experimental measurements of subjective segmentation of natural images. **a**, The participant mentally partitions the image into a pre–defined number of segments (*K* = 5 in this example) during the free-viewing epoch (not shown). Then, segmentation cues (yellow dots) are briefly shown on their own on on a gray background, and subsequently on top of the image. After the offset of the image, the participant reports whether the cues were perceived in the “same segment” or “different segments”. Reaction time (RT) is measured from image offset because participants were not allowed to respond while the image was on screen. **b**, A probabilistic segmentation map (top) is decoded from participant responses over many trials (see Methods). Each color in the legend indicates a segment, while its saturation indicates segment probability. A deterministic segmentation map (bottom) is derived by assigning the most likely segment/color to each pixel. **c**, Deterministic segmentation maps are qualitatively similar across participants (colors are arbitrary and should not be compared between maps). **d**, Reaction time histograms (*N*_*T*_ = 900 trials) for all five participants in **b**,**c**. Bin size = 48 ms

It is unclear however, if current theories can explain the spatiotemporal dynamics of segmentation of natural images, for which segments cannot be unambiguously defined. To address this challenge, we adopted a recent experimental paradigm^19^ derived from a straight-forward two–choice “same segment/different segments” task (Fig. 1). This enabled us to measure subjective spatial segmentation maps and relate reaction times to perceptual decision–making in segmentation. Our experiments revealed richer spatiotemporal dynamics than previously observed. Reaction time increased at larger distances for parts of the image perceived in the same segment, as in past reports. But, surprisingly, reaction time decreased at larger distances for parts of the image perceived in different segments.

We asked what computational principles could explain these spatiotemporal dynamics, and reasoned that inferences about image segments and features are coupled^20–23^. This is because knowledge of the segments of an image helps improve the inference of the features (e.g. each segment is expected to have a particular appearance), and, in turn, knowledge of the features helps infer the segments (e.g. similar features are expected to belong to the same segment). Therefore, inference should proceed iteratively: starting from an approximate segmentation, the inference of features is updated according to those segments, then segments are revised according to newly inferred features, and so on until the quality of inference can no longer improve. This paradigm asserts that visual inference is probabilistic and requires integrating visual inputs with prior expectations^24^ about features and segments of natural images. We refer to this theory as Iterative Bayesian Inference (IBI).

As our theoretical analysis demonstrates, IBI theory predicts that when visual inputs are consistent with prior expectations, fewer iterations are required to improve the quality of inference. This prediction correctly captures that participant reaction times increase with distance within the same segment (because nearby parts of an image are expected *a priori* to belong to the same segment), but decrease with distance between different segments (because distant parts of an image are expected to belong to different segments). In accordance with IBI, the strength of the spatiotemporal effect we observed also reflected participant choice biases measured independently of reaction time. These empirical findings could not be explained by algorithms that either ignored the prior or substituted the iterative dynamics of evidence with the simpler, linear dynamics used in the popular drift diffusion model^25,26^. Our results establish IBI as a fundamental computational principle in perceptual segmentation.

Iterative algorithms like IBI motivate functional theories of neural and perceptual dynamics^27–33^ and are abundant in both classical^34^ and modern^13,35^ machine learning. Therefore, our results have broad significance for understanding natural vision.

## Results

### Measuring perceptual segmentation of natural images.

Our experiment adopts a novel psychophysical method to measure the segmentation of entire images^19^ (Fig. 1a, details in Methods). We displayed a natural image to participants, and instructed them to partition it into a predetermined number of segments, in other words, to build a mental segmentation map. Then, in each trial, we presented two spatial cues in random locations on a gray background (250ms), and subsequently presented these same cues on the natural image (150ms). After image offset participants were asked to report with a key press, as quickly and accurately as possible, whether they perceived the two cued regions as belonging to the “same segment” or “different segments”.

From the set of responses over many trials, we decoded (see Methods) a subjective, probabilistic segmentation map for each case (*i.e.* each image for each participant, in total *N* = 58 cases across *n* = 21 participants; e.g. Fig. 1b, top). We then generated the deterministic segmentation map by assigning the most likely segment to each pixel. The segmentation maps confirmed that participants perceived meaningful visual objects as distinct segments (e.g. the elk, water, grass, and trees in Figure 1b, bottom). Maps for each image were also qualitatively consistent across participants (Fig. 1c and Supp. Fig. 1). Further visual inspection and quantification of the similarity between maps (Supp. Fig. 3) revealed fine–grained differences between individuals that captured the biases and uncertainties inherent to perceptual organization.

In addition to measuring subjective segmentation maps, we measured per–trial choice reaction times. Participant reaction time distributions were often skewed, (e.g. Fig. 1d) as is commonly observed in tasks presenting a speed–accuracy tradeoff^25^.

In summary, our empirical measurements provide a rigorous characterization of the spatial and temporal aspects of subjective perceptual segmentation of natural images.

### Spatial distance and perceived segments influence reaction times.

**Figure 2:**
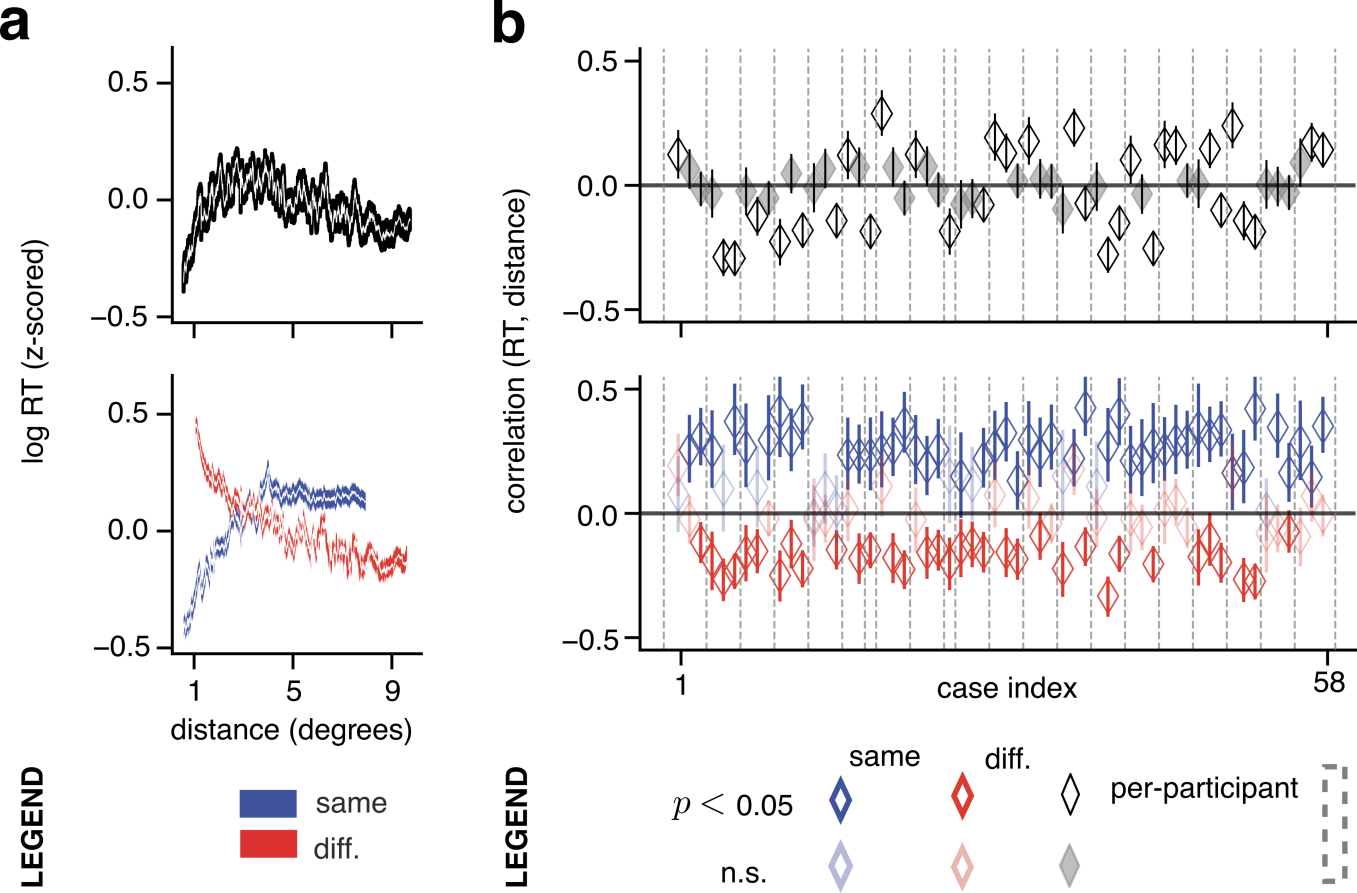
Opposite time-distance correlations within versus across segments. **a**, Aggregate across all cases (one case equals one image and participant). Top, z-scored log of reaction time plotted against distance (in degrees of visual angle) for all trials, sliding window kernel of 600 trials. White lines: mean. Shaded areas: s.e.m. Bottom, trials grouped by whether participants responded “same segment” or “different segments” in a given trial. **b**, Results per case. Top, black, unfilled diamonds indicate the Spearman correlation between reaction time and distance for cases with significant correlation (*p* < 0.05); gray, filled diamonds for cases with *p* > 0.05. Bottom, blue diamonds indicate the Spearman correlation for the “same segment” response subset, per case, while red diamonds indicate it for “different segments” response subset. Cases with *p* > 0.05 are plotted using faded colors. In both panels, error bars: 95% confidence interval; markers within vertical dashed boundaries indicate cases from a single participant.

What is the relationship between spatial and temporal aspects of perceptual segmentation? Past work that addressed this question using both synthetic^3,6,12^ and natural stimuli^9,11^ found that reaction times increased with distances between cues. Unexpectedly, we found only a weak trend between reaction time and cue–distance across all trials (Fig. 2a, top; 35,013 total trials). In individual cases, this correlation was often not significant (*p* > 0.05 for 27/58 cases) (Fig. 2b, top, faded gray diamonds). Furthermore, among significant cases, positive and negative correlations were equally common (Fig. 2b, top, black-bordered diamonds). The mean Spearman correlation across all cases was: −0.0024, s.e.m. ±0.017.

This weak time–distance correlation might appear to contradict past results. Past studies however, focused on cues placed on the same experimentally–defined object. Therefore, we repeated the correlation analysis for only the subset of trials in which participants responded “same segment” (*n* = 10,799 total trials). This subset revealed — consistent with past findings — that reaction time tended to increase with distance (Fig. 2a, bottom, blue line). In all individual cases with significant correlation (*p* < 0.05; *N* = 46/58) the correlation was positive (Fig. 2b, bottom, blue diamonds). Across all cases, the mean correlation was: 0.23, s.e.m. ±0.013.

For the complementary subset of trials in which participants responded “different segments” however, (*n* = 24,214 trials), there was a decreasing relationship between reaction time and distance (Fig. 2a, bottom, red line). In all significant cases (*N* = 34/58) the correlation was negative (Fig. 2b, bottom, red diamonds). Across all cases the mean correlation was: −0.092, s.e.m. ±0.014.

These findings explain why weak correlations were found when analyzing the complete set of trials. Opposite signs of correlations in the “same segment” and “different segments” subsets canceled out (noting that the “different segments” subset comprised 69% of all trials, but the magnitude of positive correlation was larger for trials in the “same segment” subset). This result suggests that existing theories of attentional spreading within an object paint an incomplete picture of the spatiotemporal dynamics of segmentation.

### A normative theory of segmentation dynamics.

We asked if our experimental observations could be explained by a normative theory of segmentation. The normative approach makes assumptions about the computations required to solve the segmentation problem, and then tests if algorithms that implement those computations reproduce human spatiotemporal dynamics.

Segmenting an image requires estimating the probability that each pixel belongs to any possible segment. This kind of probabilistic clustering problem is often solved with a well–established machine learning scheme that iterates between estimating the segments and estimating the visual features that comprise each segment, until convergence^36^. We hypothesized that the visual system uses a similar probabilistic clustering strategy, with a Bayesian prior that favors spatially–compact segments, which we termed IBI.

**Figure 3:**
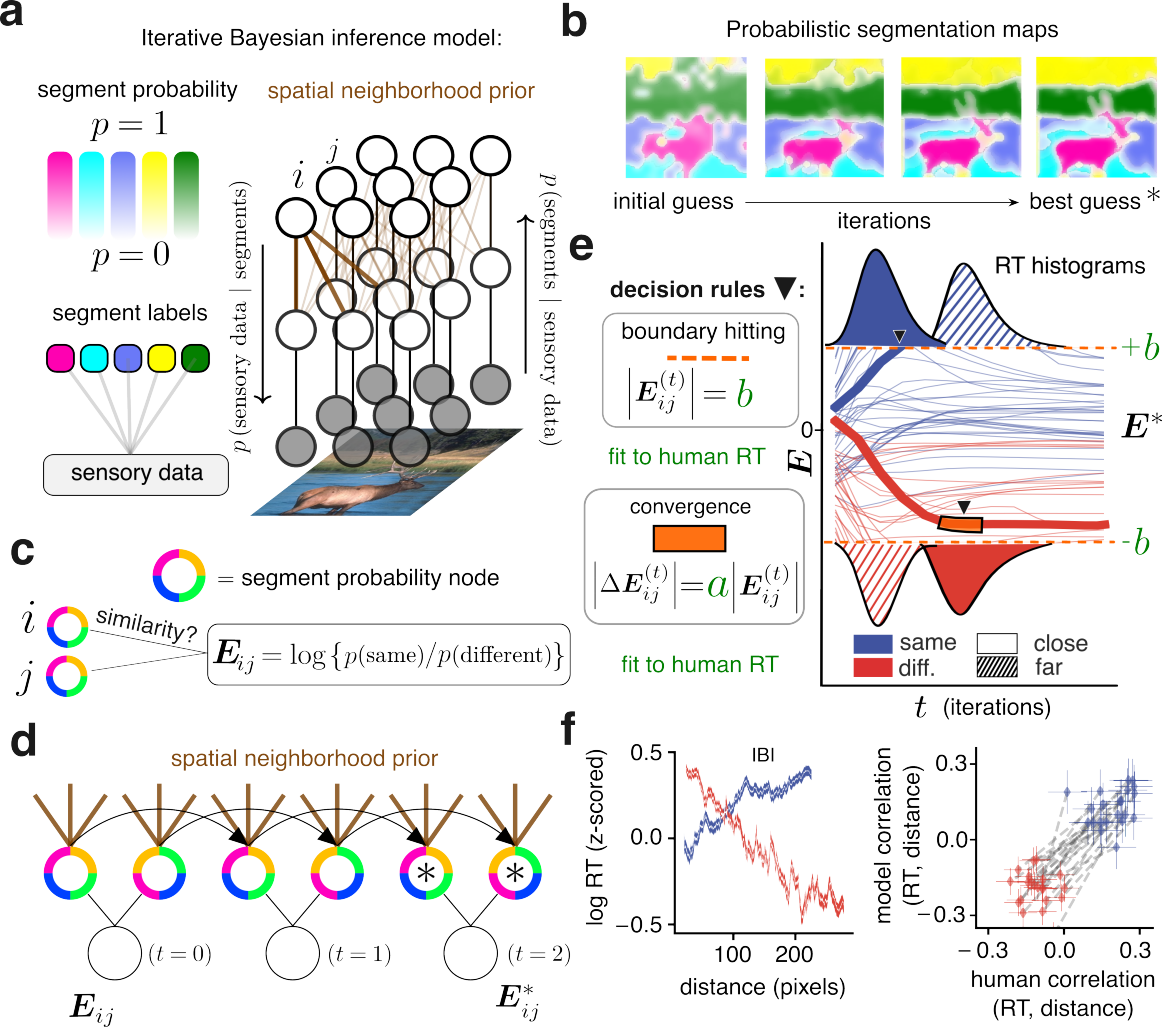
The IBI model predicts opposite time–distance correlations. **a**, Left: In IBI theory, sensory data extracted from the pixels of an image (bottom) is probabilistically assigned to a segment label (middle) with a corresponding probability (top). Colors are not semantic or ordered, as in Fig. 1b. Right: Inference of the segment assignment uses a probabilistic graphical model, with nodes indicating random variables and edges indicating probabilistic relationships. Brown edges indicate the probabilistic spatial prior. Arrows indicate information passed on the graph during two steps in which labels are inferred from the image features and segment probabilities of the previous step, and then probabilities and features are updated based on labels until convergence (see Methods section “Iterative inference” and Supp. 11D). **b**, Probabilistic segmentation maps, following the legend in **a**. The model starts from an initial guess for segment labels, which is iteratively refined to a best guess (∗) given features in the input image. **c**, The similarity between segment probabilities of two pixels is quantified by the log–probability ratio which defines the evidence E. **d**, As the model iterates (black triangular arrows), segment probabilities are updated until the best guess (∗). Evidence is computed by comparing these probabilities, which are influenced by probabilistic relationships in the spatial neighborhood prior (brown lines) at each step. **e**, Thin blue and red lines illustrate the dynamics of evidence for “same segment” and “different segments” pairs of pixels. The thick lines exemplify how a reaction time is recorded (marked by downward black triangles) when the evidence reaches a threshold value (orange dashed line) or stops increasing (orange highlights, black borders). To relate model reaction times to human reaction times two parameters (green a and b) are fit (see Methods). Top and bottom: mock RT histograms; close pairs = solid, far pairs = hatched. **f**, Left: Same conventions as in Fig. 2a, bottom, but for the IBI model, aggregated across all cases. Right: Blue diamonds indicate the time-distance correlation for the “same segment” responses subset for each participant across all images they were presented with. Red diamonds indicate the time–distance correlation for the “different segments” response subset. Gray dashed lines connect the diamonds for subsets that belong to the same participant. Error bars: 95% confidence interval.

To test this theory concretely, we adopted segmentation algorithms that implement IBI^37^ (detailed in Methods). The algorithms use latent variables that describe how the features of an input image could be organized into segments with corresponding labels and probabilities. Figure 3a depicts these variables as nodes of a graph whose edges represent probabilistic dependencies between variables. Edges that connect neighboring nodes (Fig. 3a, thick brown lines) encourage pixels in the same neighborhood to share a segment label, and therefore define the Bayesian spatial prior.

Given an input image, the algorithm starts from an initial guess for segment probability nodes and proceeds until a best guess is obtained at convergence (Fig. 3b). Dynamic segment probabilities, captured by eqs. 1 and 2 (see Methods), are therefore assigned to each node. Nodes are continually influenced by their neighbors leading to spatially heterogeneous computations that drive perceptual dynamics. For example, by inspection of Fig. 3b we can see that pixels at the center of the elk’s body (a spatially compact segment region) reached their final probability value faster than pixels at the elk’s horns (a spatially distributed segment region). This illustrates the distinctive property of IBI theory, that fewer iterations are needed to improve the quality of the inference when the visual input is consistent with the spatial prior^38^ (mathematical details in Supp. Note 11). Therefore, without fitting to data, IBI makes a qualitative prediction that captures our key experimental finding; namely, the dynamics of inference are faster for neighboring pixels that belong to the same segment, and also faster for far–removed pixels that belong to different segments, because those configurations are consistent with the IBI’s spatial prior.

### Iterative Bayesian Inference explains human perceptual segmentation dynamics.

To test if our IBI theory captures the experimental data, we mapped the dynamic segment probabilities onto a “same segment” or “different segments” choice for our experimental task. The log–probability ratio between those probabilities (termed log–odds) represents the evidence in favor of either alternative (Fig. 3c; Methods eqs. 3,4), which is updated across iterations (Fig. 3d). The model returns a reaction time either when the evidence stops changing or reaches a predetermined boundary corresponding to high certainty that one choice is better than its alternative (Fig. 3e). To fit this model to human reaction times we introduced just two parameters (Fig. 3e, green text) and initialized the model’s segmentation based on participants’ segmentation maps (see Methods).

Within the model, the dynamics of evidence and reaction times vary widely across pairs of cues. We observed that at the first iteration, due to the initial guess, evidence in favor of “same segment” choices was generally stronger for close pairs, and evidence in favor of “different segment” choices was stronger for far pairs. As the iterations progressed, the evidence for the two conditions that were consistent with the prior led to faster reaction times (solid blue and hatched red histograms in Fig. 3e) than for the other two conditions (hatched blue and solid red histograms in Fig. 3e).

We quantified this effect across cases. Similar to the human data, there was an increasing trend for the IBI model in the subset of trials in which the model responded “same segment” (Fig. 3f, left, blue line). Conversely, for the subset of trials in which the model responded “different segments” there was a decreasing trend (Fig. 3f, left, red line). The average correlation across all cases was 0.13, s.e.m. ±0.017 for the “same segment” subset versus −0.2, s.e.m. ±0.015 for the “different segments” subset. When combining all trials of both types, these trends largely canceled each other out (correlation: −0.04, s.e.m. ±0.005, across cases). Aggregated for each participant, the difference in correlations between the “same segment” and “different segments” subsets aligned with human values (Fig. 3f, right).

In summary, IBI theory reproduces the characteristic dynamics of human perceptual segmentation of natural images. It also provides a normative explanation for why these dynamics emerge from the computation of image segments.

### Stochastic linear dynamics cannot capture human perceptual segmentation dynamics.

To determine which aspects of the IBI algorithm accounted for the dynamics of human data, we compared IBI as described above to the widely adopted drift–diffusion model (DDM)^26^ of perceptual decision making. Mathematically, the DDM has stochastic linear dynamics within each trial^25^, in contrast to the nonlinear within–trial dynamics of IBI. Conceptually, iterations in IBI are used to improve the quality of the approximate inference, whereas in a DDM iterations are used to average out the noise that corrupts the evidence.

**Figure 4:**
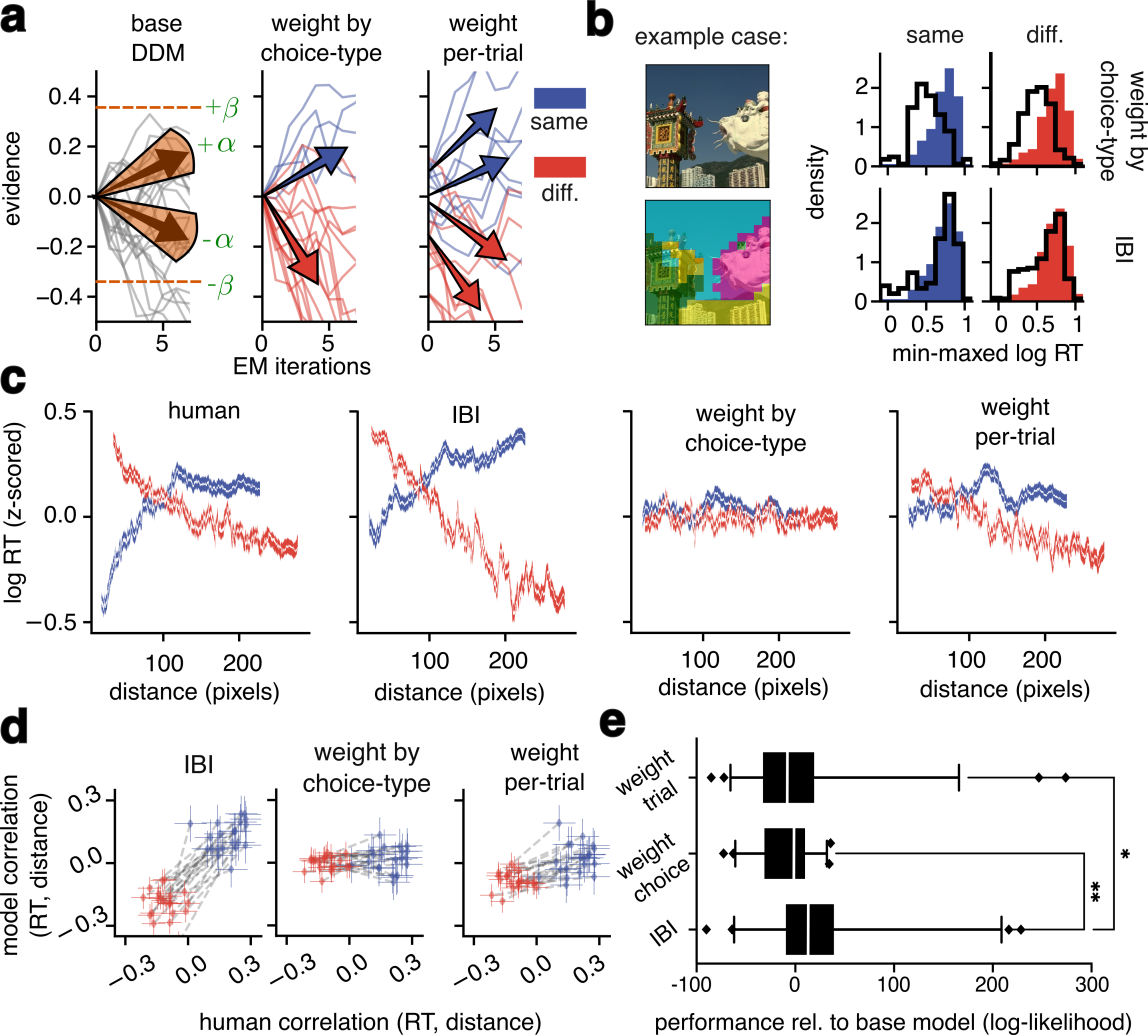
Drift-diffusion models do not fully capture spatiotemporal dynamics. **a**, Evidence traces from the base model (left), a choice-weighted DDM model (middle), and a trial-weighted DDM model (right); DDMs are as described in the main text. Traces are colored by the sign of the trace at the final iteration (not shown) for visual clarity. **b**, Histograms display the fit for the example case from the choice-weighted DDM (top row) vs. the fit from the IBI model (bottom row). Human reaction time distributions are filled in, while model reaction time distributions are overlaid as outlines, *n* =615 trials. **c**, Leftmost panel: same as Fig. 2a, bottom; other panels: same plotting conventions, but for the data generated by the model class indicated in the panel title. **d**, Leftmost panel: same as Fig. 3f, right; other panels: same plotting conventions, but for the data generated by the model class indicated in the panel title. **e**, Quantitative model performance: Log-likelihoods of all image-computable models relative to the base model. Model losses were computed using 5-fold cross-validation. Whiskers indicate 5^th^ and 95^th^ percentile values, the Kruskal-Wallis test with Dunn’s test was used for pairwise comparisons. (∗*p* < 0.03; ∗∗*p* < 0.003).

To establish a reference point, we first constructed a base DDM (Fig. 4a, left) that used two fittable parameters like the IBI model. In contrast to IBI however, the base DDM used a single evidence rate that was constant within and across trials and agnostic to image input. This model did not fit reaction time data nearly as well as IBI (the IBI performance was on average 13 times better than base DDM; Fig. 4e). One possibility is that base DDM failure was due to participants in our experiments reacting systematically faster for “different segments” choices (median decision time 480ms, 95% c.i.±4.50ms) than “same segment” choices (512ms, 95% c.i.±5.44ms; relative difference: 7%). To rule out this failure mode, we extended the base DDM by adding asymmetric evidence rates, deeming this a choice–weighted DDM (Fig. 4a, middle). The choice-weighted DDM resulted in a slightly faster median reaction time for “different segments” responses (relative difference: 0.95% in choice-weighted DDM vs. 3.43% in IBI), but did not improve histogram fits (Fig. 4b), capture distance dependence (Fig. 4c third column, 4d middle), or improve the fits quantitatively (IBI performance was on average 15 times better than the choice–weighted DDM; Fig. 4e).

The choice–weighted DDM uses a single evidence rate for all trials with the same choice type, whereas the IBI model computes the evidence per trial based on the image content at the cued locations. To compare IBI and DDM on equal footing then, we defined an image-computable, trial–weighted DDM. We used the per–pair log–odds from IBI at convergence to weigh the DDM’s evidence rate and starting–point per trial (Methods). Therefore, the trial–weighted DDM effectively included the same information about image feature similarity and the spatial prior as IBI, but maintained linear within–trial dynamics. The trial-weighted DDM improved the alignment with human time-distance correlations relative to the choice-weighted DDM (Fig. 4c,d right), but correlations remained weaker than in the actual data. Furthermore, improved alignment came at the cost of a worse quantitative fit to the empirical data (IBI performance was on average 21.2 times better than the trial–weighted DDM; Fig. 4e). Reduced trial–weighted DDMs that weighted only the drift rate or only the starting point did not improve alignment (Supp. Fig. 6). Finally, per case, the IBI model was a better fit than each of the other models in most cases (Supp. Fig. 7).

From these comparisons, we conclude that the nonlinear within–trial dynamics of IBI allow the model to capture aspects of perceptual segmentation dynamics that are not captured by the accumulation of noisy evidence at a fixed rate as conceptualized in the DDM.

### A Bayesian prior for spatial proximity modulates dynamics through two complementary mechanisms.

**Figure 5:**
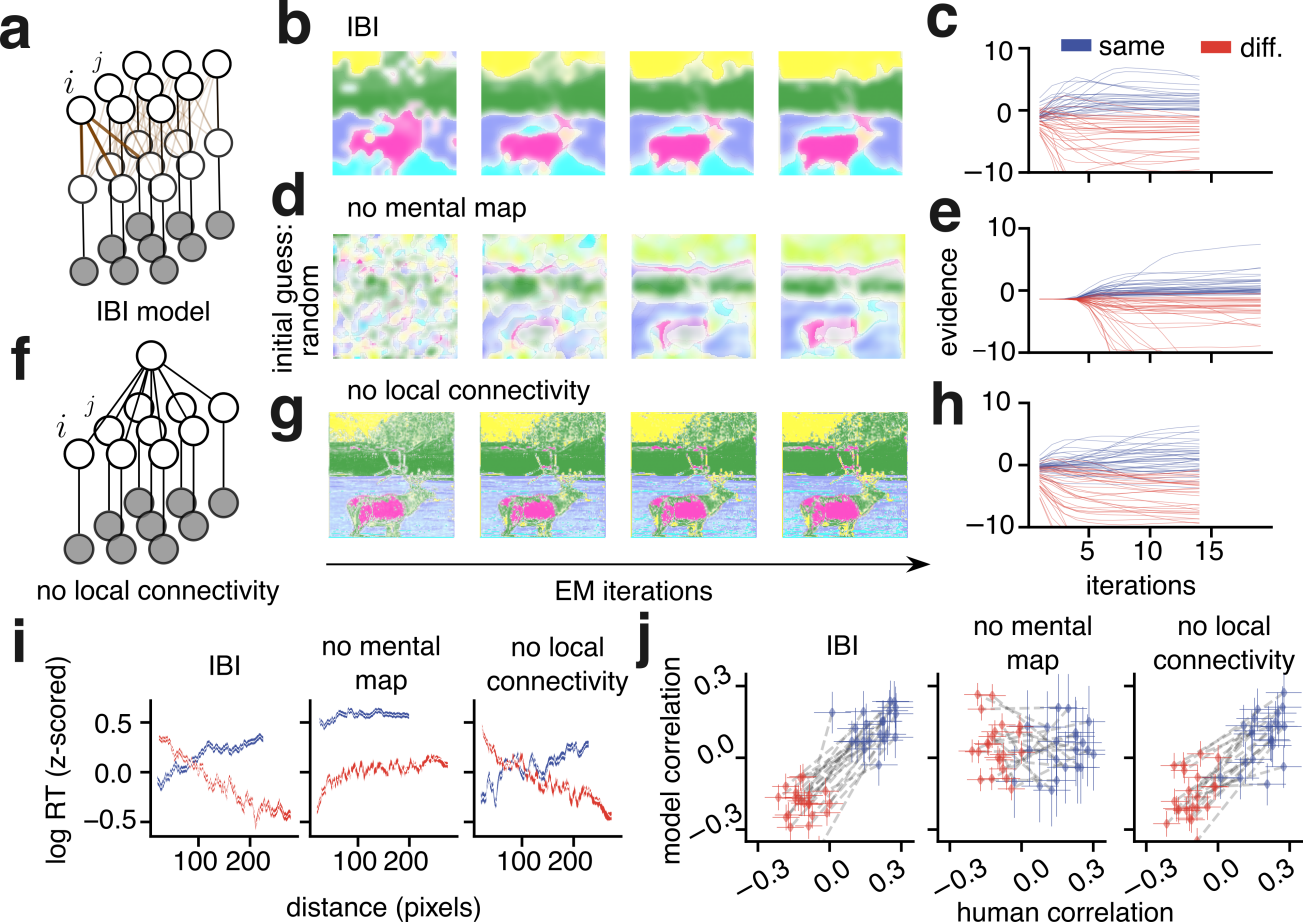
Algorithms that lack spatially compact segments do not capture human segmentation dynamics. **a**, The full graphical model (IBI, same as Fig. 3a). **b**, Initial iterations in the IBI model, for reference. **c**, Evidence traces from the IBI model, for reference. **d**, Initial iterations of the model when the initial guess is set to be uniformly random instead of using the participant’s segmentation map. **e**, Evidence traces that ensue from a random initialization. **f**, Perturbing local connectivity by replacing the top-layer of segment probability nodes with a single node, *i.e.* there is an identical prior probability of segments, for all pixels. **g**, Initial iterations of the model without local connectivity, but with the same initial guess as in panel **b**. **h**, Evidence traces that ensue from the no local connectivity model. Panels **b**,**d**,**g** use the same plotting conventions as Fig. 3b. Panels **c**,**e**,**h** use the same plotting conventions as Fig. 3e. Traces are colored by the choice made by the model in the corresponding trial. **i**,**j**, Leftmost panels: same as Fig. 3f; other panels: same plotting conventions, but for the data generated by the model class indicated in the panel title.

Having established the importance of modeling within– trial iterative dynamics, we set out to dissect the computational mechanisms through which the spatial prior influences those dynamics: first, the initial guess for the segment probabilities, which determines the starting point for dynamics; and second, graph connectivity (Fig. 5a, brown lines; u function in Methods eq. 2) which enforces the prior and consequently influences dynamics throughout the iterations.

The initial guess in IBI was based on the participants’ own segmentation map, representing the notion of a mental map. This biased the model towards segments corresponding to objects perceived by the participants, which were often spatially compact. To remove this component of the spatial prior, we used a spatially random initialization (Fig. 5d, termed ‘no mental map’). This reduced model was still able to perform the iterative computations required to update the segment probabilities, but more iterations were needed to reduce uncertainty and reach trial-by-trial decisions (compare Fig. 5e to Fig. 5c). When fitting this model to human reaction times, we observed a positive time-distance correlation for both “same segment” and “different segments” responses (Fig. 5i, middle) denoting a clear failure to capture human data (Fig. 5j, middle).

Next, we removed the graph-based local spatial prior (Fig. 5a, brown lines) by replacing all segment probability nodes with a single mean node (Fig. 5f; termed ‘no local connectivity’). The lack of local connectivity meant no information from spatial arrangement could slow down or accelerate the dynamics of evidence (Fig. 5h), and therefore decisions were made solely on the basis of image features. In order to successfully update segment probabilities, the no local connectivity model required the same initialization as the full IBI model. Nonetheless, the resulting segmentation maps were noisier than the full model and the human maps (Fig. 5g). When fit to human data, the time-distance correlations were not as aligned as the full model (note the higher negative and lower positive correlations compared to IBI and humans in Fig. 5i,j right). Furthermore, due to the noisy segmentation maps, evidence in the no local connectivity model was extremely sensitive to the precise location of the cues (see Methods and Supp. Note 11E).

In summary, perturbations of our graphical model show that the spatial prior affects within-trial dynamics through two mechanisms, and both are necessary to yield the best match to human data.

### Subjective segmentation maps reveal human spatial proximity bias.

Although the spatial prior was necessary for IBI theory to capture human segmentation dynamics, it is possible that human participants did not rely on a spatial prior for their inference of segments, and that instead the time–distance correlation reflects other unmodeled factors. To address this concern, we required a model–agnostic, experimental measurement of the spatial prior that was independent of the reaction time patterns. Specifically, we reasoned that if participants relied on a spatial prior, their choices should display characteristic proximity biases.

A proximity bias for perceptual grouping of simple parametric stimuli is well established^1^. In natural scenes however, it has not been measured before. The subjectivity of natural scene segments^19^ means that a proximity bias cannot be simply measured as a systematic deviation from an experimentally–controlled ground truth.

**Figure 6:**
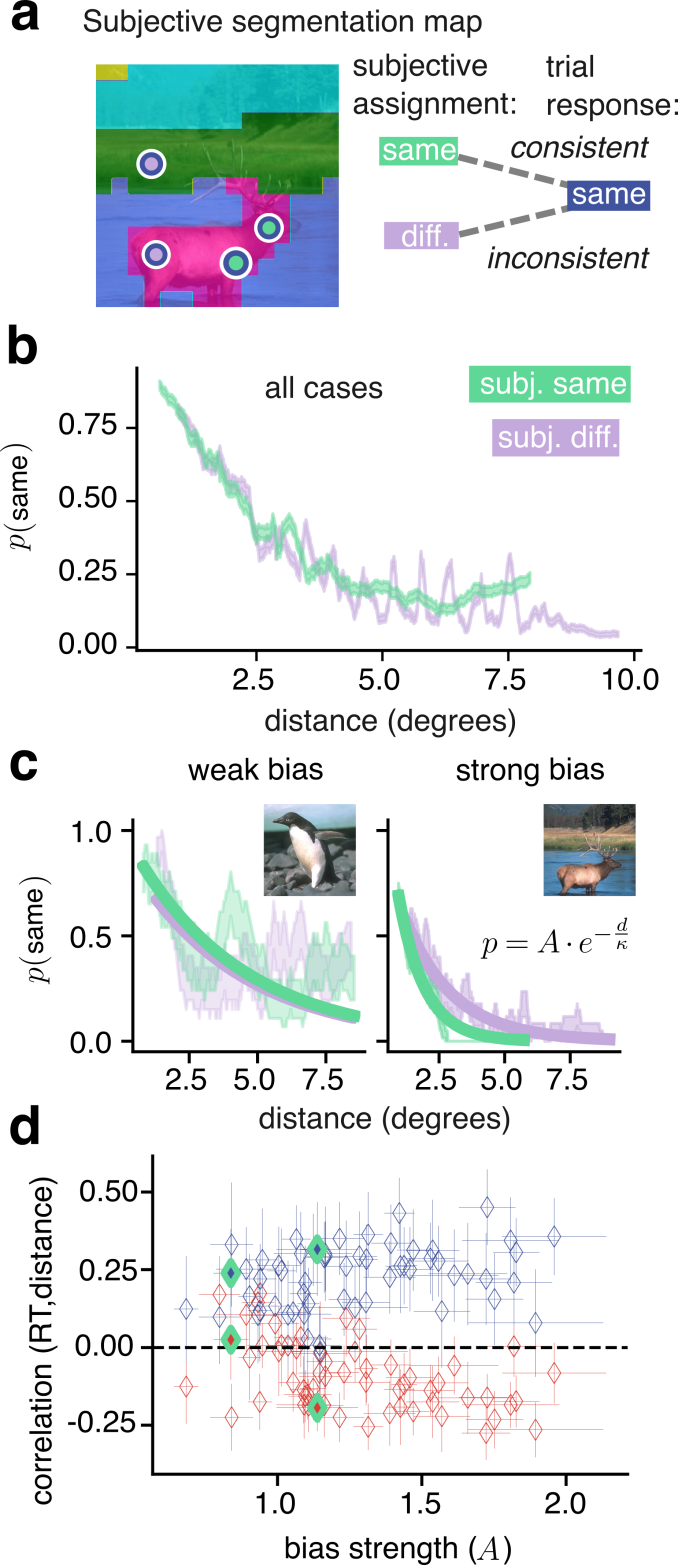
Consistency between responses and subjective maps reveals a spatial bias. **a**, A participant’s deterministic, subjective segmentation map. Markers indicate pairs of cues presented in two trials, both with “same segment” response (blue borders). The response is consistent with the subjective map in one trial (green markers) and inconsistent in the other trial (lavender markers). **b**, Aggregate across all cases and trials, the empirical probability of responding “same segment” as a function of distance (*i.e.* the proportion of trials with response “same segment” in a sliding window of 600 trials). **c** Same as panel **b** but for two unique cases, sliding windows of 20 trials (left) and 40 trials (right). Solid lines: Exponential fit to the empirical probabilities, according to the equation in the inset. **d**, Diamonds indicate correlation of “same segment” or “different segments” subsets of trials, per case, plotted against the strength of the spatial bias at each case (*i.e.* the A best fit parameter). The left and right pairs of green-bordered diamonds indicate the left and right example cases from panel **c**. Error bars indicate 95% confidence intervals.

Our experimental paradigm offers a solution. We tested for the proximity bias by studying how participants’ single–trial perceptual responses deviated from their own subjective segmentation map. In many trials (*n* =22,738/35,013; 65%), participant responses were consistent with their subjective map (e.g. Fig. 6a, green), but in a substantial minority of trials (*n* =12,275/35,013; 35%) the response was inconsistent with the map (e.g. Fig. 6a, lavender). For instance, the lavender-blue circles in Fig. 6a illustrate a trial in which two regions of the image were reported as being in the same segment, despite being labeled in different segments according to the subjective map (an inconsistent response).

We found that same–segment choices, whether consistent or not, were most frequent at short distances between cues (Fig. 6b both lines). Across all trials, the correlation between inconsistency rate and distance was −0.83, s.e.m ±0.004 (*p* < 1 × 10^−150^) for same-segment inconsistencies and 0.97, s.e.m ±0.001 (*p* < 1 × 10^−150^) for different segment inconsistencies. Note that, in the absence of inconsistent responses the green curve in Fig. 6b would remain at 1 at all distances and the lavender curve at 0. Instead, remarkably, the two curves were near identical, implying a very strong bias. Furthermore, if inconsistent responses were due purely to occasional attention lapses or motor errors, their proportion would be independent of the spatial separation between cues.

These results support the existence of the hypothesized spatial proximity bias for perceptual segmentation of natural images.

### Human proximity bias reflects a Bayesian prior for segmentation.

A general, normative prediction of our IBI theory is that when the prior is stronger, so are its effects on perceptual dynamics. This led us to two specific predictions.

First, if the measured proximity bias reflects a Bayesian prior, we should observe that the magnitude of correlation between reaction time and distance is larger for cases with larger bias. We took advantage of the fact that, although the proximity bias was a robust feature of our data, its magnitude varied substantially. We quantified this by fitting a decaying exponential function to the proportion of “same segment” responses, as illustrated in Figure 6c (*R*^2^ > 0.5 for 53/58 and 43/58 cases, respectively for trials with ground–truth “same segment” and “different segments”). Larger values of the amplitude parameter A and smaller values of the spatial decay constant *κ* correspond to a stronger proximity bias. We found that when the bias was stronger, the time–distance correlations had larger magnitude (correlation of bias versus the magnitude of time–distance correlation differences: 0.57, 95% c.i. ± 0.17; *p* < 1 × 10^−5^), consistent with rational use of the Bayesian prior (Fig. 6d and Supp. Fig. 8).

**Figure 7:**
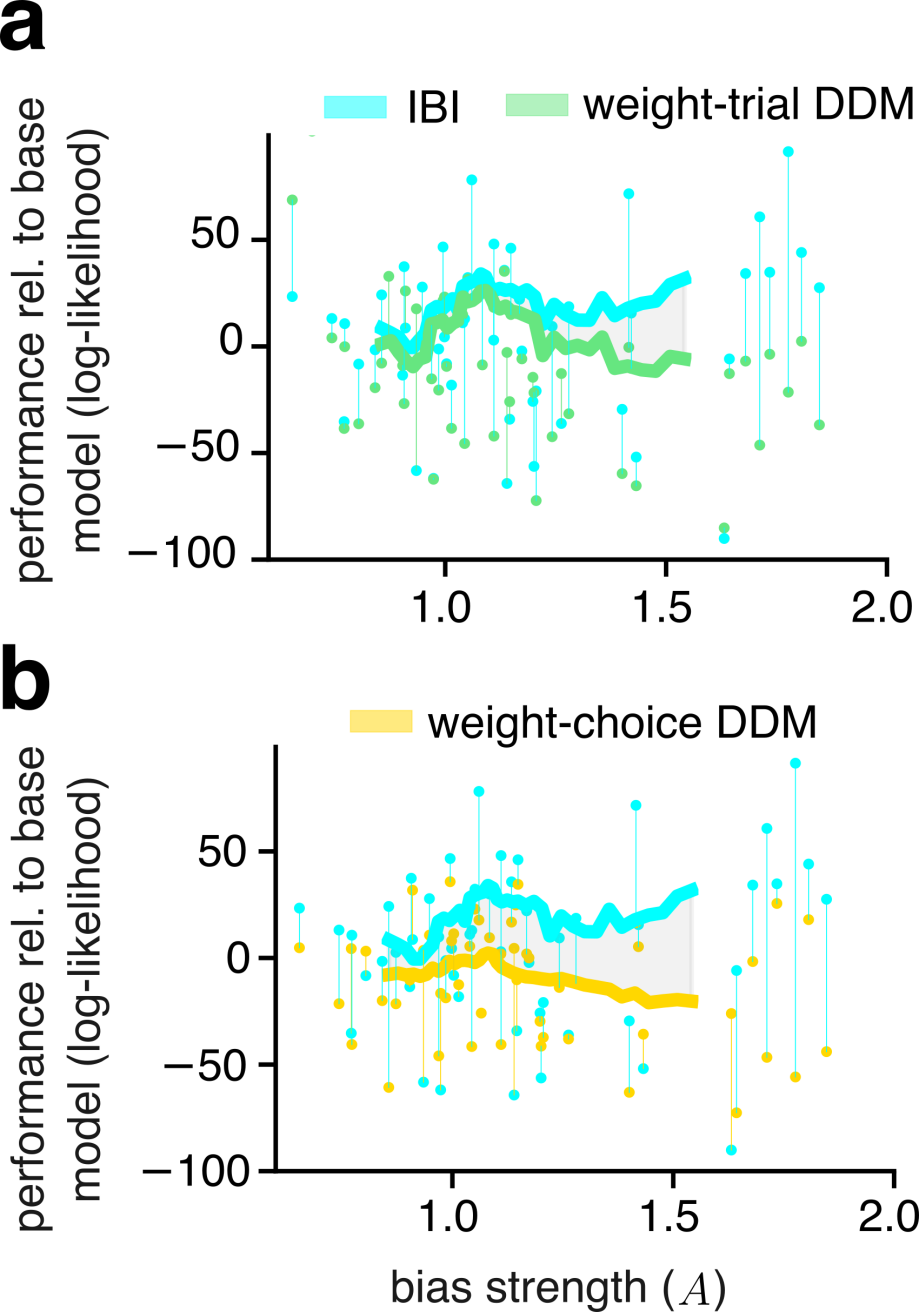
Cases with stronger human bias distinguish models with versus without spatial prior. **a**, Cross-validated model performance relative to the base model (defined as in Fig. 4e) as a function of bias strength. Each dot denotes model performance for one case. Each vertical line connects the performance of the IBI model (cyan) and a trial-weighted DDM (green). Thick lines: running average of the models performance with a sliding window of 15 cases. The shaded gray area indicates the difference in average performance between the two models. **b**, same as panel **a** but comparing IBI versus the choice-weighted DDM. In **b**, 6 cases with data points outside y-axis limits were excised to improve readability (but were included in the running average).

The second prediction follows from the fact that the prior used by the IBI model was never fit to the human data, it was fixed in the model through local connectivity. Therefore, the difference in fit quality between models that operate with a spatial prior and models that do not, should increase for cases with larger bias. Consistent with this prediction, we found that for cases with higher proximity bias, IBI improved against the choice–weighted DDM (Fig. 7a) which has no spatial prior. Furthermore, IBI also improved against the trial–weighted DDM (Fig. 7b) in which the spatial prior is only applied once to the drift and starting–point, but not applied at every iteration.

These results confirm that the proximity bias we measured experimentally reflects a Bayesian spatial prior for segmentation of natural images, and that the prior influences perceptual dynamics as predicted by IBI theory.

## Discussion

Current understanding of perceptual segmentation and its neural basis^12^ relies strongly on the classical observation that the larger the distance between two locations in an image, the longer it takes to make sameness judgments^3–5,7,9,10^. We have shown that in natural image segmentation, this classical trend in perceptual dynamics is often inverted when the two locations belong to different segments (Fig. 2). We have proposed a theory that predicts both the classical effect and its inverse (Fig. 3), and explains the variability observed across individuals (Fig. 6,7). In our theory, dynamics emerge from iteration between inferring the features of the visual input and inferring the segments that those features belong to, with a Bayesian prior that favors spatially compact segments. Ignoring these structured dynamics of sensory inference, as is common in traditional models of perceptual decision–making, reduces the ability to capture empirical observations (Fig. 4). Our results indicate that iterative Bayesian inference may be a fundamental computational principle for organizing natural visual inputs.

To reconcile our findings with classical experiments on segmentation dynamics, we highlight important differences in experimental design and in the underlying theoretical framework. The first experiment to report increasing reaction times with distance used a curve tracing task^3^. In that study, distance was defined by the length of curve between the two cues, and therefore distance could not be defined for cues on different curves. In one study that modified the task to define distance both within and across segments, the trends that emerged were similar to our findings^4^. However, motivated by the within-object focus of the sequential attention spreading hypothesis, major subsequent studies emphasized dynamics within the same curve^5–7^ or within the same object^9,10^.

Like attention spreading, our IBI theory also centers on the dynamics of the visual representation of segments. However, in addition to considering within-object dynamics, IBI posits that the spatial prior acts across the entire visual input. This motivated us to compare dynamics within and between segments. Furthermore, as we measured subjective segmentation maps perceived by participants instead of experimentally defined segments, we tested the role of the prior in a way that previous studies could not. Namely, we have quantified the extent to which this spatial prior leads to a Gestalt-like proximity bias for each individual case. While such a bias was known for simplified and parametric stimuli^1^, we have provided the first measurements in natural images (Fig. 6).

Several computational models have been developed to account for classical findings on segmentation dynamics^6,10,11,13,14^ but their applications to natural images have been limited. The ‘growth–cone’ model^6^ worked well for outline images but failed for natural images^10^, likely because it was not image-computable (*i.e.* it did not consider the image features). Other image-computable models were either trained only on object outlines^13^ and thus not applicable to natural images, or used hand–crafted feature detectors that are not effective for natural images^14^, with one recent exception^11^. However, all of the aforementioned models produce positive correlations between reaction time and distance regardless of choice type, which is not compatible with our data.

Our IBI theory offers a probabilistic account of perceptual decision–making with natural images. According to probabilistic coding paradigms, neural representations of sensory variables are best understood as approximate Bayesian inference^39^ that is optimized to natural input statistics^32,40–42^. Our contributions expand the scope of those principles. First, our model is compatible with Bayesian visual–cortical representation models and substantially extends them to build richer representations of natural images: it performs joint inference of features and segments of entire images via recurrent information flow on a retinotopically organized graph. Second, our analysis reveals that the iterative dynamics required to compute joint inferences are the main determinant of the timing of perceptual segmentation decisions.

It is important to note that our results do not argue against evidence accumulation and related DDMs as fundamental in decision making. In fact, that framework provides a normative basis for how our model makes decisions using the log–odds. However, most DDM formulations are disconnected from the time–course of inferring the evidence itself. We have proposed IBI as a general framework to address this shortcoming and demonstrated that IBI models can indeed be used to define image–computable DDMs. This enables single–trial predictions for both model classes and facilitates meaningful model comparison. Model comparison demonstrated that a DDM’s ability to capture spatiotemporal dynamics of segmentation improves when it includes image information. However, we also found that within–trial inferential dynamics—which emerge naturally from IBI— are necessary to fully account for the data (although there remains room for improving the quantitative fit of IBI models to the data; Supp. 14). There are modified formulations of DDMs that capture non–linear within– trial dynamics, for instance with time-dependent decision boundaries or drift^31,43,44^. To capture our data however, those DDMs would need to be parametrized as a function of both choice type and distance between cues. Rather than deriving new ad–hoc parameterizations of DDMs, our modeling demonstrates the advantage of considering how the visual system represents natural images. We thus show that within–trial dynamics emerge normatively from a theory that encompasses sensory inference and decision making.

Within–trial dynamics in our model also lead to predictions for neural activity. Single–neuron dynamics in V1 align well with the positive correlation between decision time and distance^12^. Our novel finding, that this correlation can be reversed, raises the question of whether a corresponding signature exists in early visual cortex. In addition, correlates of the spatial prior may be evident in large–scale dynamics of spontaneous^45^ and evoked^15^ activity, reflecting the two computational mechanisms needed to integrate the spatial prior in IBI (Fig. 5).

Although we have focused on segmentation, the insights from IBI theory are likely relevant for modeling perceptual and neural dynamics with natural stimuli more broadly given that ecological priors abound in perception^46,47^, and that natural stimuli often engage non– trivial sensory cortical dynamics^45,48^.

## Methods

### Experimental procedures.

#### Participants.

We recruited 21 participants (11 female; age range 16–30 years) with normal or corrected to normal vision. Participants were naive to the task, and they (or their guardians, for minors) gave signed consent to participate in the experiments, and were compensated according to institutional guidelines. The study was approved by the Internal Review Board of Albert Einstein College of Medicine and Montefiore Medical Center (IRB number: 2019–10297).

#### Task details.

We used the experimental paradigm and task design validated in our earlier work^19^. We first provided verbal instructions followed by a brief training session (25 trials). Each participant then completed three “blocks” (we term all trials for one image a block) separated by short breaks. At the beginning of each block, participants were presented with an image and instructed to mentally segment it with a predetermined number of segments, K, ranging from 3 to 5 (blocks were ordered pseudo–randomly). Next, the image was presented on the screen and disappeared after 5 seconds. A prompt instructed the participant to press a key to start the task. In each trial, we displayed two cues (red circles with 1–degree diameter) on a gray screen for 250ms and then the same two cues superimposed on the image for 150ms. Participants were not asked to maintain fixation throughout the trial but eye movements were likely minimal during the brief image presentation time. After the image and cues disappeared, participants were prompted to report whether the two cued locations belonged to the same segment or different segments. The prompt remained on the screen until participants responded with a key press. Participants were instructed to answer as quickly and accurately as possible. The following trial started after a delay of 50–250ms.

The center of each cue was selected from coordinates defined by a square grid of size 15 × 15. The grid covered the entire area of the image at lower resolution. Participants’ subjective segmentation maps were decoded at this resolution. For brevity, we refer to each pair of cues with the indices (i, j) where each i or j represents a 2D coordinate. Cues were selected pseudo–randomly with the constraint that the pair was unique in each trial. In each block, we presented the minimum number of trials required to decode the segmentation maps at the desired resolution (a small subset of all possible pairs of cues^19^) that depends on K: *N*_*T*_ = 450, 675, 900 trials for *K* = 3, 4, 5, respectively.

#### Visual stimuli and presentation.

We selected 12 natural images manually from the Berkeley Segmentation Database^49^, and cropped them to patches of size 256 × 256 pixels (see Supp. 1, 3 for image ensemble). Each image was prescribed a single value for K, and segmented by 4–6 distinct participants. Images were presented on a gamma–calibrated LCD monitor. Participants used a chin rest to maintain a viewing distance of either 67cm or 96cm and we rescaled image size to span 8.7 × 8.7 degrees of visual angle. We combined data for the two viewing distances as no systematic differences were observed.

#### Data preprocessing.

In each trial we recorded the binary response $R_{ij}$ and the corresponding reaction time $t_{ij}$ (*i.e.* the time between image offset and key press). Before analyzing reaction times, we excluded trials with $t_{ij}$ larger than the 90^th^ percentile (those outlier trials may correspond to attentional lapses). We verified that our main results held without this exclusion (Supp. Fig. 9), and when we controlled for sequential across–trial effects (Supp. Fig. 15). We then z-scored log transformed reaction times separately for each case (*i.e.* one image and one participant) so that the reaction time trends could be meaningfully compared across participants and images.

#### Decoding segmentation maps.

We decoded the subjective segmentation of an image from the set of responses to all pairs presented in the experimental block as detailed in our previous work^19^. Specifically, we estimated the probability that any location on a 15 × 15 grid is assigned to any of the K segments, and termed this grid of probabilities a probabilistic segmentation map (e.g. Fig. 1b, top). This was done by numerically minimizing the squared difference between the measured response $R_{ij}$ and the probability that the pair i, j is in the same segment, averaged over all pairs (we also confirmed that this approach did not generate signal from noise, by shuffling participant responses across pairs; Supp. Fig. 2). From the probabilistic segmentation maps, we computed a deterministic segmentation map by labeling each location on the grid with the segment that had highest probability (e.g. Fig. 1b, bottom). Lastly, we up–sampled the maps to match the resolution of the experimental image.

### Iterative Bayesian Inference models.

#### Background.

Our IBI algorithm was based on flexible probabilistic mixture models (termed FlexMM^37^). We describe here the four main stages of the algorithm (further details are in Supplement 11).

#### Feature extraction.

Given an input image, we first extracted features by passing the image through a deep neural network pretrained for object recognition^50^. The feature space encodes more abstract information than image pixel intensity, such as edge orientation or texture. We used early-to-intermediate convolutional layers of the network because these have shown alignment with neural activity in visual cortex^51^. Next, we applied Principal Component Analysis to reduce the dimensionality of each layer and obtained a 12-dimensional representation $x_{i}$ at each pixel i (mathematical details in Supp. 11A).

#### Generative model.

FlexMM assumes a generative model in which each observation $x_{i}$ depends probabilistically on a set of latent variables (*i.e.* variables inferred from observations rather than directly observed), as summarized by the graph of Fig. 3a in which nodes represent variables and edges represent probabilistic dependencies between variables^34^. In the graph, each gray node corresponds to the image features $x_{i}$ for pixel i (Fig. 3a, bottom layer). The variable $c_{i}$ represents the segment label that the features of pixel i belong to (Fig. 3a, middle layer). The variable $\pi_{i}$ (Fig. 3a, top layer) is a vector of segment probabilities, *i.e.* the probability that the features of pixel i belongs to any segment label. Given an input image, these probabilities $\pi_{i}$ are what we aim to infer to obtain the probabilistic segmentation map of the image.

Following previous work relating image statistics to visual–cortical activity^32,40–42^, we assumed that all the features $x_{i}$ within a given segment with label k follow a Student-t distribution with parameters collectively denoted $\theta_{k}$ (see Supp. 11B for the specific parametrization). The spatial prior is enforced by introducing additional edges between $\pi_{i}$ and the c variables in a local neighborhood (Fig. 3a, brown edges) encouraging pixels in the same neighborhood to share the same segment label.

#### Iterative inference with a spatial prior.

From the generative model of FlexMM, we were interested in computing the probabilistic segmentation map π of any given input image. Because exact computation of these probabilities is not tractable, FlexMM uses an iterative approximation scheme that extends the popular ExpectationMaximization (EM) algorithm for probabilistic clustering, to account for the spatial prior as follows. EM iterates to find the values of π (the organization of segments) and θ (the organization of features within each segment) that increase the likelihood of the observations x. At iteration t, the Expectation step uses the previously inferred values of the $\pi^{\left(t-1\right)}$ and $\theta^{\left(t-1\right)}$ to update the approximation of the posterior probability for c, denoted $\gamma^{\left(t\right)}$. Then, using the current $\gamma^{\left(t\right)}$, the Maximization step updates $\pi^{\left(t\right)}$ and $\theta^{\left(t\right)}$ to their new best value, while incorporating the spatial prior by considering the current values of γ in a spatial neighborhood (full derivation in^37^ and Supp. 11D):

(1) $$\gamma_{i,k}^{\left(t\right)}≐p\left(c_{i}=k|x;\pi^{\left(t-1\right)},\theta^{\left(t-1\right)}\right)\;\;\;\propto \pi_{i,k}^{\left(t-1\right)}\cdot \text{Student}\left(x_{i};\theta_{k}^{\left(t-1\right)}\right)$$

(2) $$\pi_{i,k}^{\left(t\right)}=\frac{u\left(\gamma_{1,k}^{\left(t\right)},\ldots \gamma_{H\times W,k}^{\left(t\right)}\right)}{\sum_{l}^{K}u\left(\gamma_{1,l}^{\left(t\right)},\ldots ,\gamma_{H\times W,l}^{\left(t\right)}\right)}$$

FlexMM implements the spatial prior through the function for u which must be linear but is otherwise unconstrained. We used a discrete convolution with a 2–dimensional Gaussian kernel of finite size denoted by H×W (H=W in our formulation). Following from eqs. 1 and 2, the spatial prior was introduced iteratively; the probability of the segment of each pixel was updated by averaging the previously inferred segment probabilities within a spatial neighborhood. Similarly, to further improve segmentation performance, the update of π at one feature layer took into account also the previously inferred segment probabilities in neighboring layers (see Supp. 11D.5 for details).

In addition, we introduced the spatial prior also through the initial guess $\pi^{\left(0\right)}$ for each image and participant, by sampling from the participant’s subjective segmentation map which was often comprised of spatially compact segments (see Supp. 11D.6 for details).

In FlexMM, convergence of the likelihood is guaranteed^37^ and therefore the dynamics of the probabilistic segmentation map are well defined. EM convergence is typically defined globally for the image (*i.e.* on the likelihood summed over all pixels). Yet, the segment probabilities of all pixels do not converge at the same time (mathematical details in Supplement 11D). As explained next, this is important when applying FlexMM to our experimental task.

#### Decision rules for IBI.

Because individual pixels displayed unique dynamics, so too did pairs of pixels (see traces in Figs. 3e; 5c,e,h). Our decision rule was based on comparing the probability that a pair of pixels i, j are in the same segment (denoted $\pi_{ij}$) versus in different segments $\left(1-\pi_{ij}\right)$. At each iteration, these quantities were computed from the per–pixel probabilities and their log–ratio was used as evidence in favor of one option or the other:

(3) $$\pi_{ij}^{\left(t\right)}=\sum_{k}^{K}\pi_{i,k}^{\left(t\right)}\pi_{j,k}^{\left(t\right)}$$

(4) $$E_{ij}(t)=\left[\log\left(\pi_{ij}^{\left(t\right)}\right)-\log\left(1-\pi_{ij}^{\left(t\right)}\right)\right]$$

Positive values of $E_{ij}$ imply evidence in favor of “same segment” and negative values in favor of “different segments”. The model could report a decision at the first iteration for which the evidence reached a boundary b for “same segment” or −b for “different segments”, which was a fittable parameter. We also included a second decision rule, to account for the fact that, for some pairs, $\pi_{ij}$ might converge to a value that does not reach the boundary. Specifically, the model could report a decision when the change in evidence between consecutive iterations $\left|\Delta E_{ij}^{\left(t\right)}\right|$ became smaller than the quantity $a\left|E_{ij}^{\left(t\right)}\right|$, where a is the second fittable parameter. We recorded the model decision and corresponding reaction time at the first iteration in which either rule was satisfied (models that used only a single rule performed worse; Supp. Table 3, Supp. Fig. 10).

### Drift Diffusion Models.

#### Background.

We compared the within-trial dynamics of IBI against an alternative with strong support in classical decision-making literature, drift diffusion models (DDMs)^25,26^. We briefly review the theory before describing the bespoke, image-computable DDMs introduced in this work.

The DDM models an optimal observer—that is an observer who responds the fastest given an acceptable maximum error rate. Sequential samples of evidence, which are corrupted by independent samples of noise, are optimally integrated over time using the sequential probability ratio test, *i.e.* each new sample updates the ratio, L(t), between the log–likelihood of each alternative hypothesis. DDMs assume that each sample of evidence in a given experimental condition contributes the same amount on average. Based on this assumption, the log– likelihood ratio can be parametrized in continuous time as:

(5) L(t)=z+mt+ηW(t)

Where z is a starting point, m the mean rate of evidence (drift) and W(t) is a Wiener diffusion process such that: dW∼N(0,1), where the variance of the diffusion process is scaled by η. In a DDM, dynamics are unique across trials because drift is perturbed by independent random draws from the Wiener process. The objective of a DDM is not to make per-trial predictions of human choices or reaction times, but to match the characteristically skewed reaction time distributions that arise from perceptual decision-making tasks^25^.

#### Base DDM.

We first fit a base DDM model which was not image-computable. Defining the base DDM through equation 5, we set z=0 and fit m directly to the human data per-case without any consideration of image features. η was set heuristically and fixed across cases. This procedure is in line with the typical application of DDMs in which the drift rate m is not directly encoded in stimulus information, but decoded from fitting to empirical data^26^.

#### Image-computable *DDM.*

Image-computable DDMs, hereafter referred to as weighted DDMs, used the segment probabilities from IBI to define the per-trial DDM parameters. In our trial-weighted DDMs we substituted m and z with the value $E_{ij}^{\left(t^{*}\right)}/t^{*}$ computed from eq. 4, obtained at time $t^{*}$ which is the time of IBI’s global convergence to a final map (Fig. 4a, right; further details in Supp. 12). Reduced variants in which we substituted only one of m or z performed poorly (Supplementary Note 6). In our choice-weighted DDMs, we set z=0 and simply averaged the per-trial values $E_{ij}^{\left(t^{*}\right)}/t^{*}$ over each choice type grouped by the final map at time $t^{*}$ (Fig. 4a, middle).

One interpretation of using the IBI evidence at convergence to define the DDM parameters, is assuming that the time required for sensory inference is much less than the time required for integrative decision–making subject to noise (see Supp. 12B). In other words, weighted DDMs and IBI capture the same feature and spatial information to use as evidence, but DDMs assume linear within–trial dynamics while ignoring nonlinear dynamics.

#### Decision rules for DDM.

Because DDMs assume that evidence grows at a constant rate on average, only one stopping rule is needed for when evidence hits the boundary, β. We distinguish the boundary for DDM models as β because it is fit from scratch using no information from the boundary for IBI models b. In addition, we also fit a global gain parameter α that scales the evidence for all trials.

### Model fitting and comparison.

#### Preprocessing.

Before model fitting, we normalized reaction times per case between 0 (shortest reaction time) and 1 (longest), separately for the human data and for each model.

#### Parameter fitting.

All IBI and DDM models used two free parameters, a and b or α and β. Best fit parameters were found by maximizing the likelihood that the observed human reaction times come from the distribution of model reaction times. While some DDM’s leverage an analytical solution for this likelihood, we wanted to standardize the fitting procedure across IBI and DDMs models. Therefore, we used the empirical cumulative distribution to compute the likelihood instead. We computed such likelihoods separately for the trials in which humans chose “same segment” vs. trials in which the humans chose “different segments”, and used the negative of the sum of those two terms as the cost function to be minimized (equivalent to a maximum-likelihood approach; details in Supp. 13). This cost function was not differentiable with respect to the parameters, so we used Markov-Chain Monte-Carlo (MCMC) global minimization methods (see Supp.13 for details). Note that model parameters were not optimized to capture trial-by-trial human responses, yet model responses were largely consistent with the human subjective maps thanks to the initialization (Supp. Fig. 4).

#### Model comparison.

For fair comparison across models we used 5–fold cross validation per case. Models were fit using trials in a training split, and then applied to predict reaction times in unseen trials that were part of a test split. All reported results are from applications to test splits *i.e.*, unseen data.

#### Implementation details.

To add stability to all models we computed the reaction time for each pair ij over 10 pseudo-coordinates that were randomly selected to be within the spatial neighborhood of pixel i and j. Using 10 pseudo-coordinates provided 100 pseudopairs that ensured small floating-point differences from pixel to pixel did not impact model results. The code is computationally inexpensive, and can be run locally.

### Statistical analysis.

To plot the z-scored log of reaction time versus distance, we sorted reaction times by distance, and applied a sliding window kernel to add smoothing and elucidate trends. When comparing median reaction times for the “same segment” and “different segments” subsets, we used bootstrapping with 9999 resamples to compute 95% confidence intervals.

We quantified the relation between reaction time and distance with the Spearman correlation and we used parametric statistics to compute confidence intervals (similar results were obtained with Pearson correlation; Supp. 5).

For comparing relative likelihood distributions from each model type, we first confirmed that models were sufficiently different from each other using the nonparametric Kruskal-Wallis test. For pairwise comparisons we used Dunn’s test with a significance level of α=0.05; p–values were corrected for multiple comparisons.

To fit exponential curves for quantifying spatial biases (Fig. 6) we used a nonlinear least-squares regression. Parameters A and κ were optimized to fit a curve with the parametrization p=A⋅exp(−d/κ), where p is the probability of responding “same segment” and d is the independent variable (distance between pixels). During fitting, we applied the bounds [0.5,4.3] (units in degrees) to κ. These were chosen as reasonable lower and upper bounds for the space constant given the size of the image. 95% confidence intervals for fit parameters were computed using two standard deviations of optimal parameter estimates.

## Supplementary Material

Supplement 1
