# Supplementary material for: Natural scene segmentation dynamics reveal iterative Bayesian inference
Source: bioRxiv. 2026 Feb 2:2026.01.30.702842. Preprint. [Version 1] doi: 10.64898/2026.01.30.702842 (PMC12889477; doi:10.64898/2026.01.30.702842)
Supplement: Supplement 1 [file NIHPP2026.01.30.702842v1-supplement-1.pdf]

bioRxiv preprint doi: https://doi.org/10.64898/2026.01.30.702842; this version posted February 2, 2026. The copyright holder for this preprint (which was not certified by peer review) is the author/funder, who has granted bioRxiv a license to display the preprint in perpetuity. It is made available under aCC-BY 4.0 International license.

Supplementary Information

1 Subjective segmentation maps - all images **17**

2 Control decoding of subjective segmentation maps - all images, randomized responses **18**

3 Similarity between subjective segmentation maps - aRI comparison **19**

4 Consistency between subjective maps and single-trial responses and between model and human responses - confusion matrices **20**

5 Correlation between reaction times and distance – details **21**

6 Reduced trial-weighted DDMs **22**

7 Per-case model comparison **23**

8 Bias against κ **24**

9 Results without trial exclusion **25**

10 Comparison of model performance with different decision rules **26**

11 Mathematical details **27**
- A Feature extraction . . . 27
  - A.1 Feature selection . . . 27
- B Gaussian scale mixtures . . . 28
  - B.1 Mixtures of Gaussian Scale Mixtures . . . 28
- C Defining the graphical model for FlexMM . . . 28
- D Learning and inference dynamics . . . 29
  - D.1 EM as recurrent message passing . . . 29
  - D.2 EM as variational inference . . . 31
  - D.3 Per-pixel EM . . . 32
  - D.4 EM as gradient ascent . . . 32
  - D.5 Deep spatial prior . . . 34
  - D.6 EM initial guess . . . 34
- E Graphical model perturbations . . . 34
  - E.1 No local connectivity model . . . 34
  - E.2 No mental map model . . . 36
- F Model convergence . . . 36
  - F.1 The iterative timescale . . . 36
- G Evidence for “same segment” . . . 36

12 Drift Diffusion modeling **37**
- A Sequential log-probability ratio test . . . 37
- B Iterative and Integrative timescales . . . 37
- C Types of DDMs . . . 38

13 Parameter fitting **40**

14 IBI model extensions **41**

15 Reaction times decrease throughout the experimental block **42**

bioRxiv preprint doi: https://doi.org/10.64898/2026.01.30.702842; this version posted February 2, 2026. The copyright holder for this preprint (which was not certified by peer review) is the author/funder, who has granted bioRxiv a license to display the preprint in perpetuity. It is made available under aCC-BY 4.0 International license.

Supplementary Note 1: Subjective segmentation maps - all images

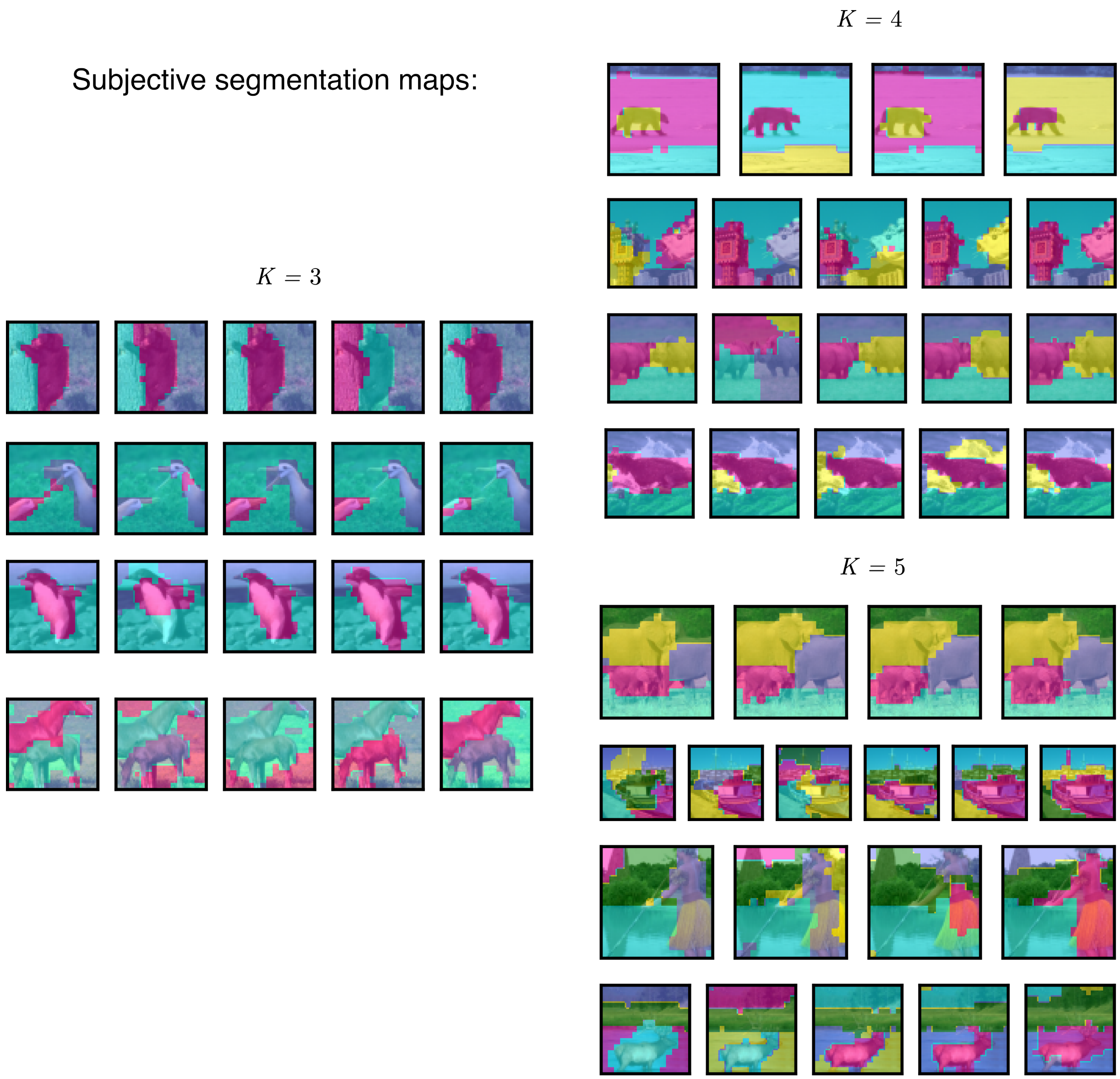

Supplementary Figure 1: Subjective segmentation maps for all images and participants. Each panel is the segmentation map for one case (one image by one participant). Images are organized by the number of segments requested, K. Each region in a 15×15 grid is assigned the most–likely segment. As in Figure 1b,c.

bioRxiv preprint doi: https://doi.org/10.64898/2026.01.30.702842; this version posted February 2, 2026. The copyright holder for this preprint (which was not certified by peer review) is the author/funder, who has granted bioRxiv a license to display the preprint in perpetuity. It is made available under aCC-BY 4.0 International license.

Supplementary Note 2: Control decoding of subjective segmentation maps - all images, randomized responses

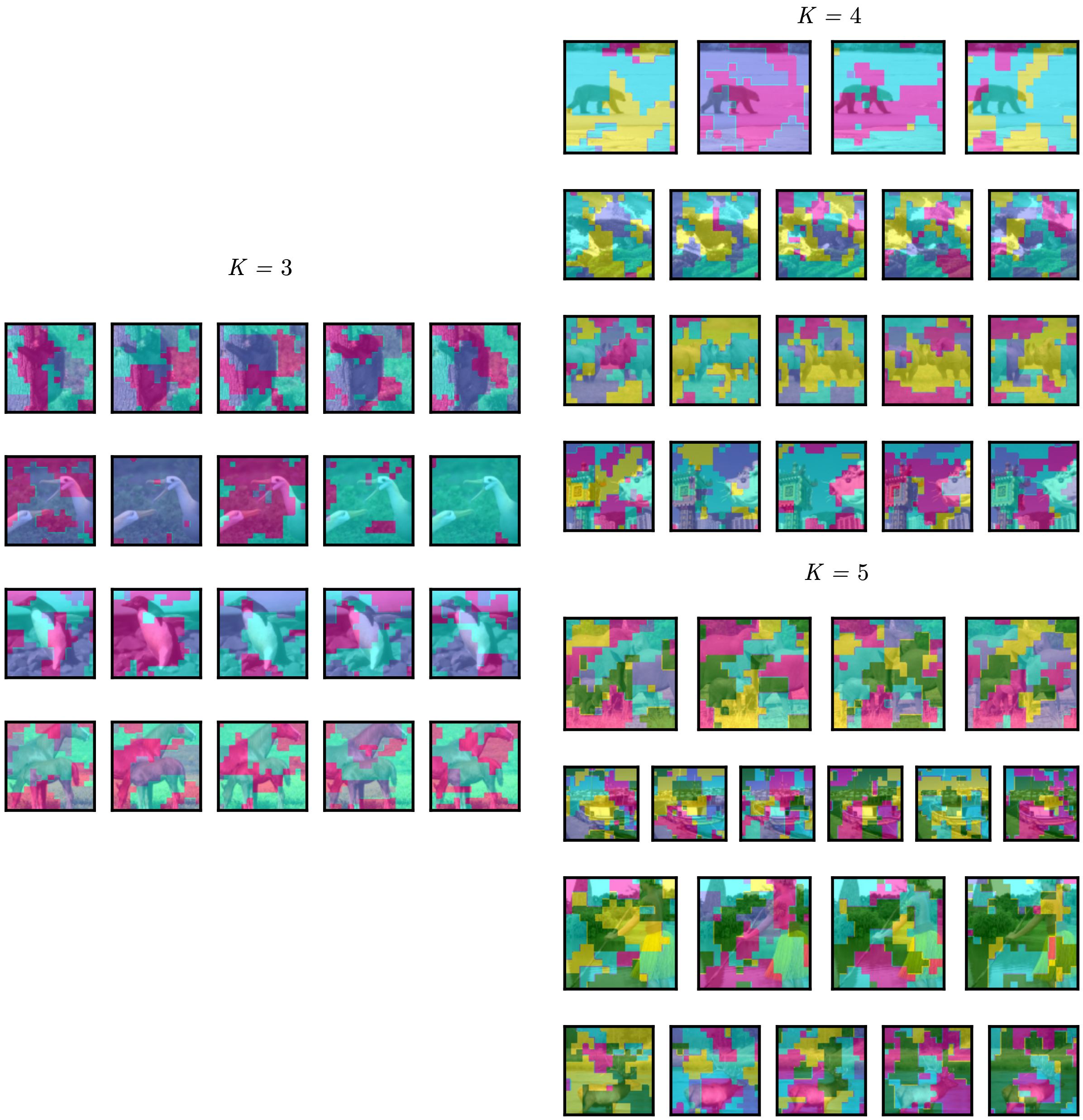

Supplementary Figure 2: Decoded subjective segmentation maps with participant responses shuffled across trials. We devised a procedure to verify that the decoding algorithm could not hallucinate segments when the binary choice data did not contain segment-related information. To do so, we randomly shuffled the order of the responses across trials, so that each response was not aligned with the actual cue pair shown in the corresponding trial, but it was instead aligned with a different cue pair. This figure shows that, in that case, the maps produced by the decoder were not meaningfully related to the image contents.

bioRxiv preprint doi: https://doi.org/10.64898/2026.01.30.702842; this version posted February 2, 2026. The copyright holder for this preprint (which was not certified by peer review) is the author/funder, who has granted bioRxiv a license to display the preprint in perpetuity. It is made available under aCC-BY 4.0 International license.

Supplementary Note 3: Similarity between subjective segmentation maps - aRI comparison

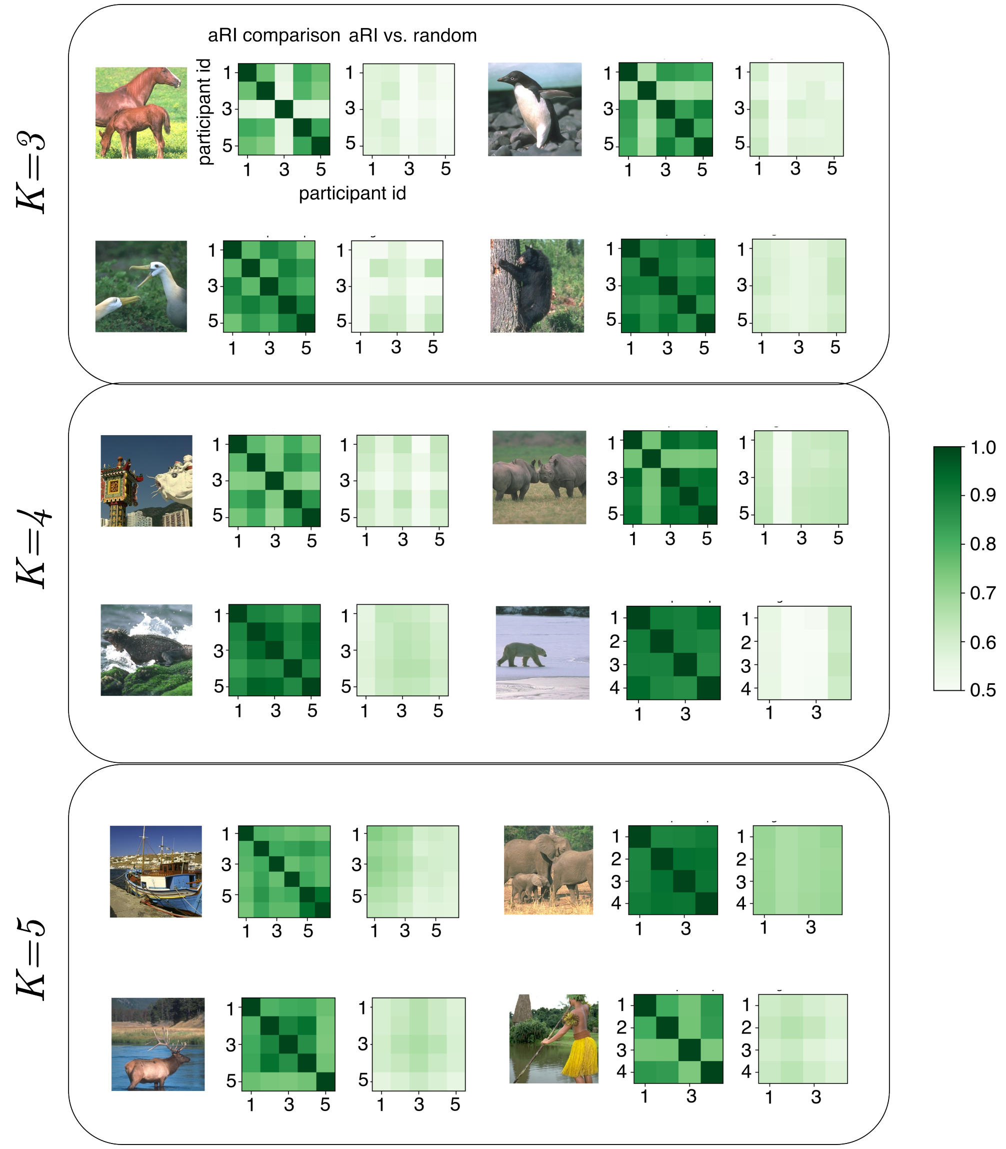

Supplementary Figure 3: Map similarity across participants. We use the adjusted Rand Index (aRI) [1] to compare the similarity of subjective maps for each image across participants. For each image, the matrix on the left represents the comparison between all pairs of participants that segmented a particular image, where each entry is the aRI comparing their maps. aRI is a symmetric measurement so the matrices are symmetric across the diagonal. The diagonal indicates a map compared to itself; by definition the value is 1. To obtain a baseline value for similarities in maps that arise from the decoding process itself, we compared the aRIs of the maps in Supp. Fig. 2 (note that the participant ids are not meaningful in the right-hand side matrix, as all participant responses are shuffled)

We use the adjusted Rand Index (aRI) [1] to quantify similarity. The aRI is a metric for comparing two clusterings that is agnostic to the cluster labels. In terms of the segmentation maps in Supp. Fig. 1, the aRI does not consider the segment colors. Instead the aRI considers all possible pairwise assignments within one map versus all possible pairwise assignments in another. A value of 1 means that all pairwise assignments are equivalent. A value of 0.5 means that, when adjusted for chance, only half of the two map's pairwise assignments agree with each other.

bioRxiv preprint doi: https://doi.org/10.64898/2026.01.30.702842; this version posted February 2, 2026. The copyright holder for this preprint (which was not certified by peer review) is the author/funder, who has granted bioRxiv a license to display the preprint in perpetuity. It is made available under aCC-BY 4.0 International license.

Supplementary Note 4: Consistency between subjective maps and single-trial responses and between model and human responses - confusion matrices

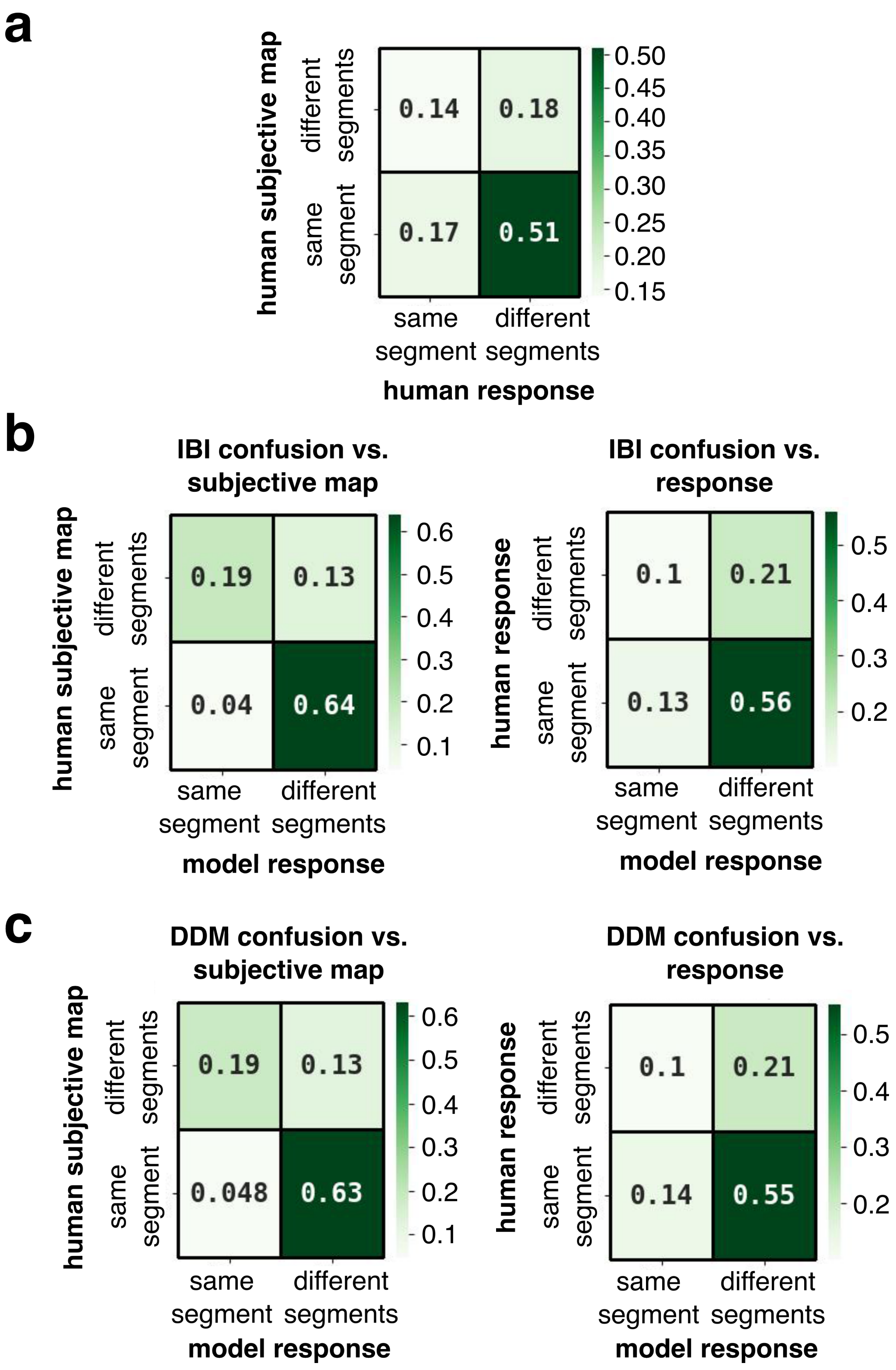

Supplementary Figure 4: Confusion matrices. **a**, Consistency *i.e.* confusion between the subjective map and single-trial responses. **b**, Consistency of IBI with the subjective map (left) and with single-trial responses (right). **c**, Here, "DDM" refers to the choice-weighted DDM. Same layout as panel **b**. The value reported in the confusion matrices are computed from all experimental trials, *i.e.* aggregated across cases.

bioRxiv preprint doi: https://doi.org/10.64898/2026.01.30.702842; this version posted February 2, 2026. The copyright holder for this preprint (which was not certified by peer review) is the author/funder, who has granted bioRxiv a license to display the preprint in perpetuity. It is made available under aCC-BY 4.0 International license.

Supplementary Note 5: Correlation between reaction times and distance – details

In the main text, we report results using the Spearman correlation coefficient (r_s) for two main reasons. First, the pre–existing literature makes no prediction that reaction time as a function of distance between cues should be linear, only increasing, and the Pearson correlation coefficient (r) tends to penalize non–linear increasing functions[2]. Second, the output of our model is based on the iteration index which is an ordinal value, not a countable passing of time like human reaction times. Therefore, it is more appropriate to use rank correlation metrics such as the Spearman correlation.

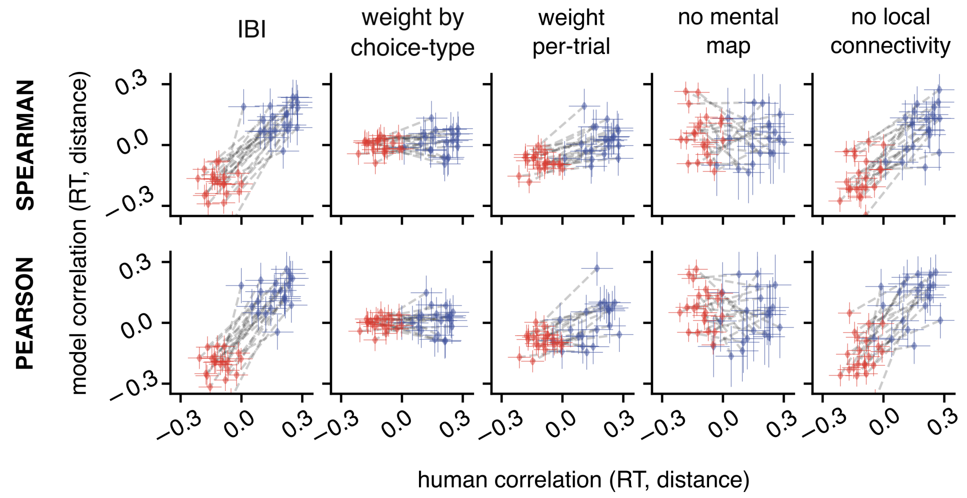

Supplementary Figure 5: Comparing Spearman and Pearson correlation coefficients. Top row: Model correlations versus human correlations using the Spearman correlation coefficient (same plotting conventions as Fig. 3f, right; Fig. 4d; Fig. 5j). Bottom row: Model correlations versus human using the Pearson correlation coefficient (same plotting conventions as Fig. 3f, right; Fig. 4d; Fig. 5j)

Nevertheless, we verified that our results do not change with the use of r compared to r_s (Supp. Fig. 5). In fact, using r better differentiates the full IBI model from reduced versions in some cases (notably the no local connectivity model). To construct confidence intervals for the two metrics we used the following formulas. For the 95% confidence interval for r, where N_T is the number of trials:

$$\mathrm{CI}_r = \tanh\left[\arctan(r) \pm \sqrt{\frac{1}{N_T-3}} \times 1.96\right] \qquad \textbf{(S1)}$$

And for r_s, where $\mathcal{T}(p, N_T-2)$ is the percentile point function for percentile p in a Student-T distribution with N_T-2 degrees of freedom.

$$\mathrm{CI}_{r_s} = \tanh\left[\arctan(r_s) \pm \sqrt{\frac{1+{r_s}^2/2}{N_T-3}} \times \mathcal{T}(0.975, N_T-2)\right] \qquad \textbf{(S2)}$$

These equations and their justification can be found in Ruscio[2].

bioRxiv preprint doi: https://doi.org/10.64898/2026.01.30.702842; this version posted February 2, 2026. The copyright holder for this preprint (which was not certified by peer review) is the author/funder, who has granted bioRxiv a license to display the preprint in perpetuity. It is made available under aCC-BY 4.0 International license.

Supplementary Note 6: Reduced trial-weighted DDMs

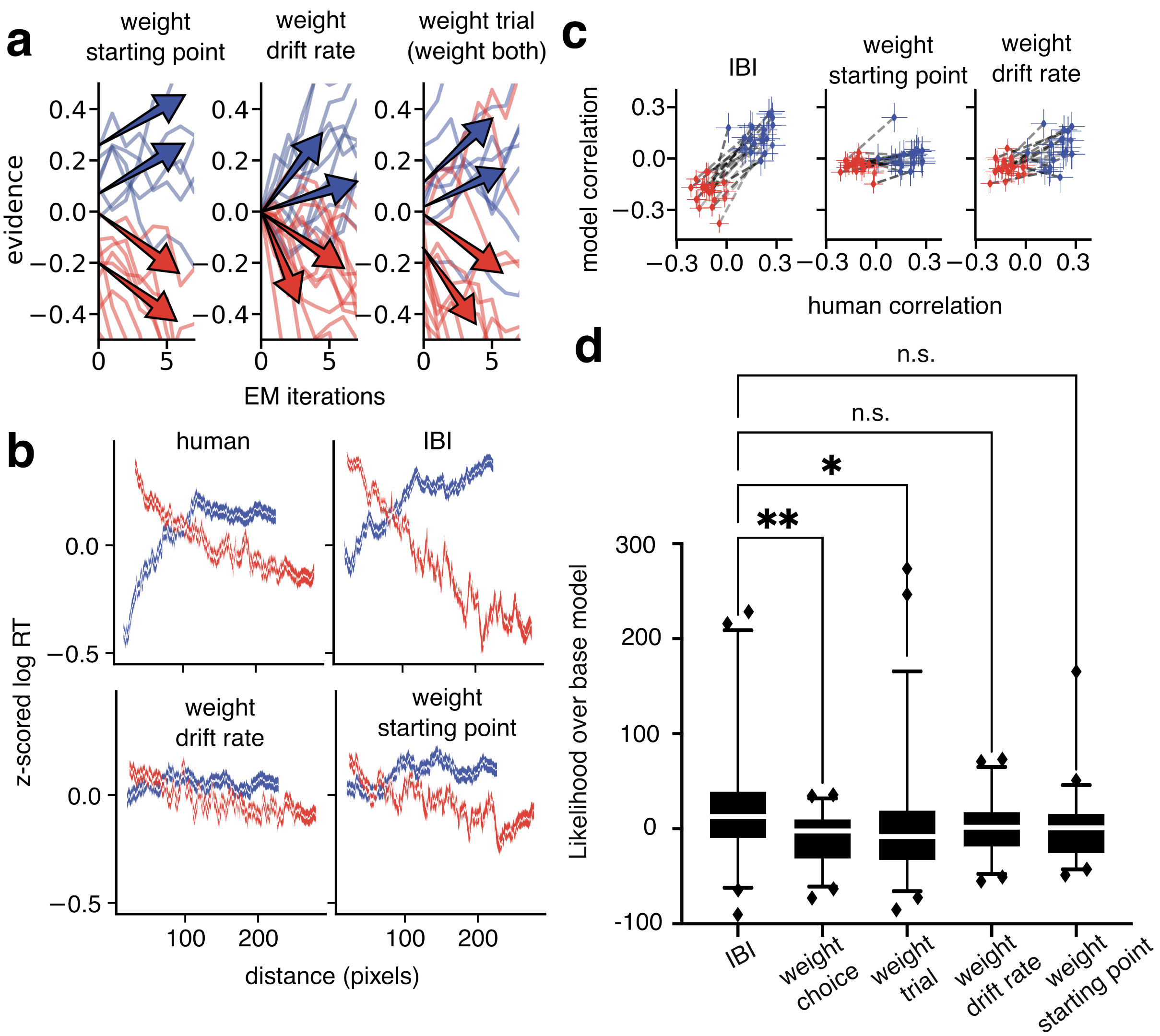

Supplementary Figure 6: Reduced trial–weighted DDMs do not capture human correlations. **a**, Same plotting conventions as in Fig. 4a. **b**, Same plotting conventions as in Fig. 4c. **c**, Same plotting conventions as in Fig. 4d. **d**, Same plotting conventions as in Fig. 4e.

Reduced trial–weighted models are those in which only one of z or m from Methods eq. 5 is weighted. Weighing only z is referred to as the weight starting point model and weighing only m is referred to as the weight drift rate model. See Supp. 12 for precise definitions. Correlation plots in Supp. Fig. 6 are flatter than the corresponding plot for the weight per–trial model in Fig. 4d.

bioRxiv preprint doi: https://doi.org/10.64898/2026.01.30.702842; this version posted February 2, 2026. The copyright holder for this preprint (which was not certified by peer review) is the author/funder, who has granted bioRxiv a license to display the preprint in perpetuity. It is made available under aCC-BY 4.0 International license.

Supplementary Note 7: Per-case model comparison

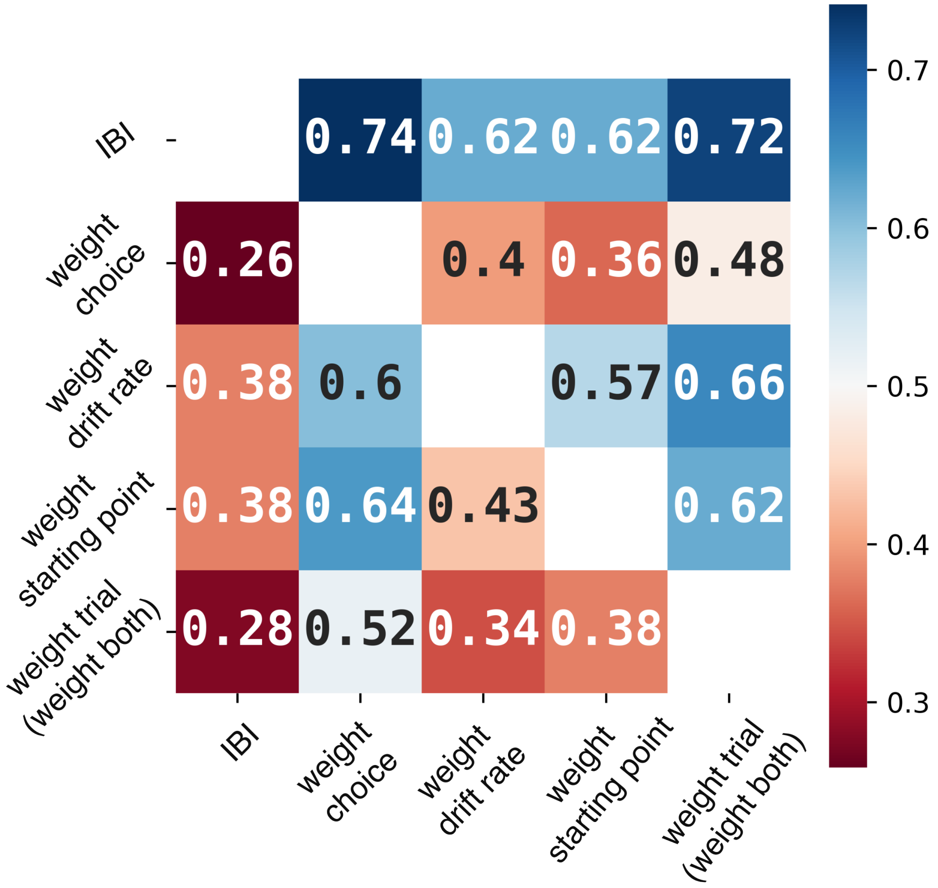

Supplementary Figure 7: Model comparison, all cases. Annotations in each cell indicate the proportion of cases in which the model specified in the row label is better than the model specified in the column label (e.g. IBI is better than choice-weighted DDM in 74% of cases, out of 58 total.)

bioRxiv preprint doi: https://doi.org/10.64898/2026.01.30.702842; this version posted February 2, 2026. The copyright holder for this preprint (which was not certified by peer review) is the author/funder, who has granted bioRxiv a license to display the preprint in perpetuity. It is made available under aCC-BY 4.0 International license.

Supplementary Note 8: Bias against κ

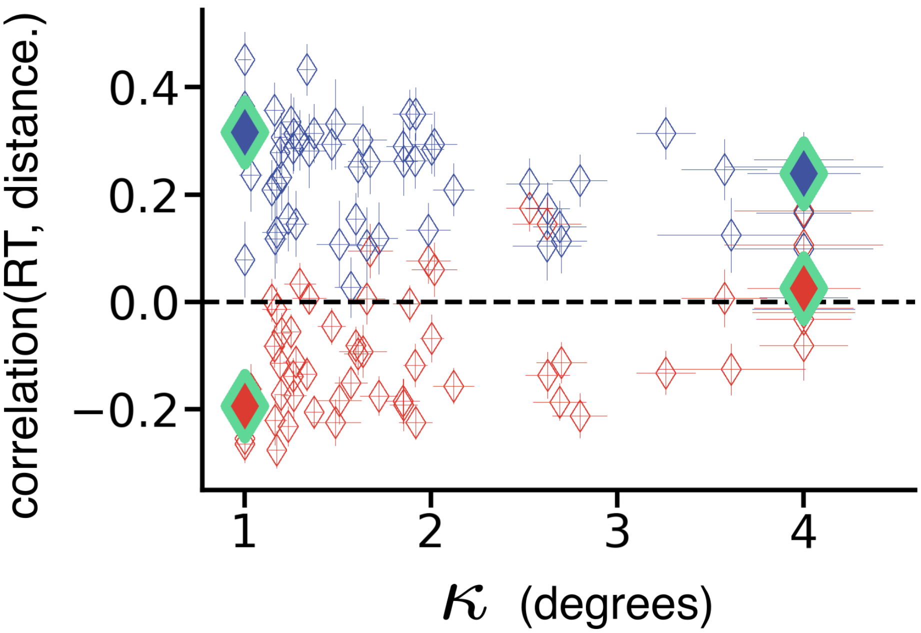

Supplementary Figure 8: A stronger bias leads to higher correlations. Same as Fig. 6d, as a function of the space constant of the exponential fit, κ, instead of a function of the amplitude A. Green diamonds refer to the same examples as in Fig. 6c. Note that smaller values of κ correspond to stronger spatial bias.

bioRxiv preprint doi: https://doi.org/10.64898/2026.01.30.702842; this version posted February 2, 2026. The copyright holder for this preprint (which was not certified by peer review) is the author/funder, who has granted bioRxiv a license to display the preprint in perpetuity. It is made available under aCC-BY 4.0 International license.

Supplementary Note 9: Results without trial exclusion

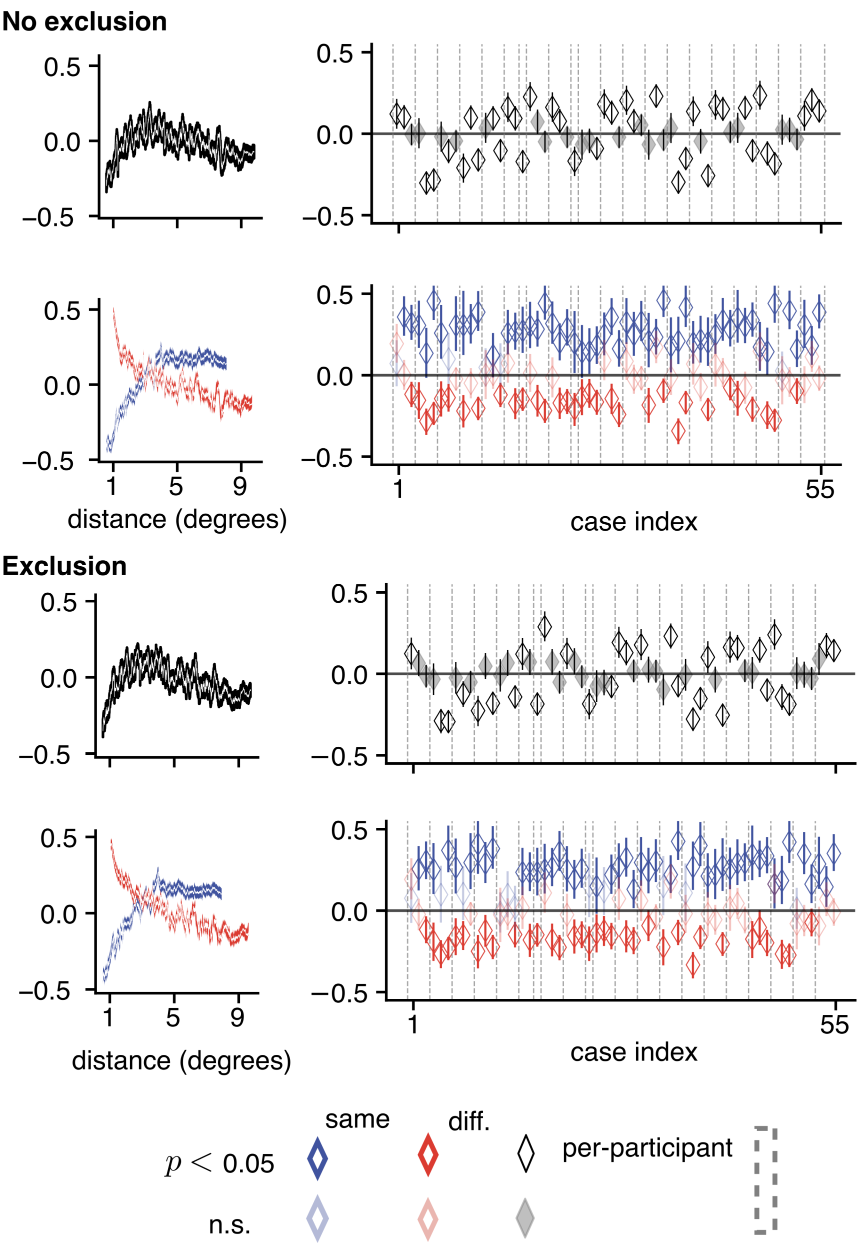

Supplementary Figure 9: Trial exclusion does not affect results. **a,c** Same as Fig. 2. **b,d** Same as Fig. 2, but without excluding the trials with the 10% slowest RT as detailed in Methods. The results are qualitatively indistinguishable.

bioRxiv preprint doi: https://doi.org/10.64898/2026.01.30.702842; this version posted February 2, 2026. The copyright holder for this preprint (which was not certified by peer review) is the author/funder, who has granted bioRxiv a license to display the preprint in perpetuity. It is made available under aCC-BY 4.0 International license.

Supplementary Note 10: Comparison of model performance with different decision rules

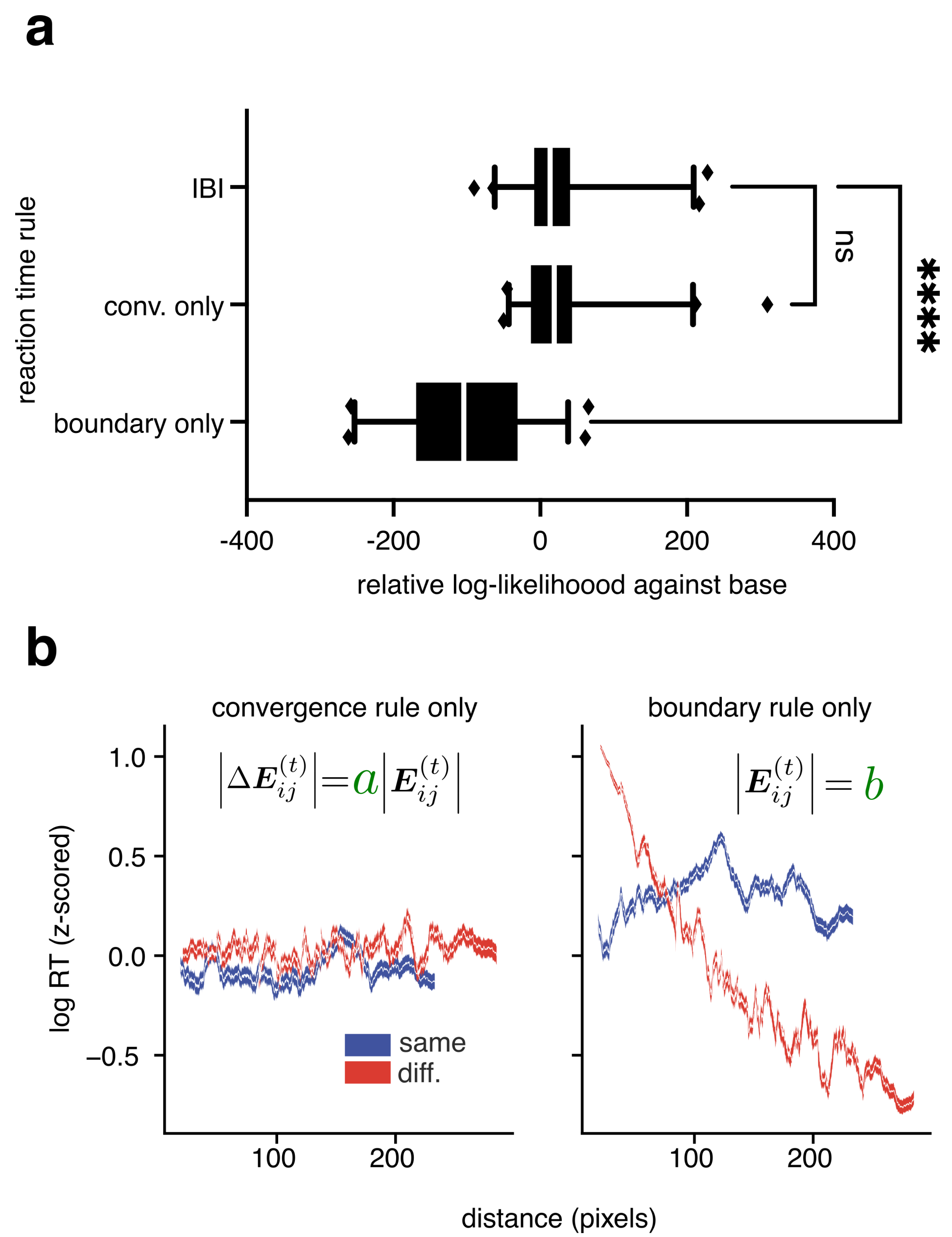

Supplementary Figure 10: Comparing decision rules a, Same plotting conventions as Fig. 4e. **b** Same plotting conventions as Fig. 3f.

To implement the convergence rule, we smoothed the evidence using a small sliding window (3 iterations) when taking the derivative. We also ensured that convergence was not transient by prescribing that the rule needed to be followed for at least 3 iterations.

The convergence rule is largely responsible for matching model distributions to human RT distributions (Fig. 10a). However, we can observe that the match to human time-distance correlations arises only when a boundary is used for the decision rule (Fig. 10b), implying that natural scene segmentation decisions involve a threshold on certainty.

bioRxiv preprint doi: https://doi.org/10.64898/2026.01.30.702842; this version posted February 2, 2026. The copyright holder for this preprint (which was not certified by peer review) is the author/funder, who has granted bioRxiv a license to display the preprint in perpetuity. It is made available under aCC-BY 4.0 International license.

Supplementary Note 11: Mathematical details

In this section, we derive the update rules presented in equations 1,2, and give an overview of the normative theory underlying our model (subsections A-D). We also discuss reduced versions of the IBI model (subsection E), and how the model produces evidence for a "same segment" or "different segments" decision (subsections F,G).

A. Feature extraction. We begin with the sensory input, an RGB image $\mathbf{I}$ with height h and width w (in pixels). The first step is to extract features from the RGB image, which we accomplish using the deep convolutional architecture VGG-19[3]. Deep convolutions remap RGB values onto high-dimensional feature vectors that have been shown to be useful as predictors of neural activity in early cortex[4]. Deep neural networks have also been shown to align with the hierarchical human visual system[5]. In principle however, any shallow or deep feature extraction applied to RGB images can be used with the probabilistic inference used in proceeding steps.

–

Notation:

- $\mathbf{I} \in \mathbb{R}^{h\times w\times 3}$: $\mathbf{I}$ is a tensor of shape $(h,w,3)$ *e.g.* an RGB image.
- $l \in [1,16]$: the layer of the neural network.
- $\mathbf{A}^{[l-1]}$: the activation from the previous layer of the neural network.
- $\mathbf{W}^{[l]}$: a square sliding window kernel with size 3 and feature dimension $F^{[l]}$, at the current layer. E.g. for layer 1: $F^{[1]} = 64$, $\mathbf{W}^{[1]} \in \mathbb{R}^{3\times 3\times 3\times 64}$ 3.
- $\mathbf{Z}^{[l]} \in \mathbb{R}^{h\times w\times F^{[l]}}$: the output of convolution operation
- $b^{[l]} \in \mathbb{R}^{F^{[l]}}$: the bias at a given layer

$$\mathbf{I} = \mathbf{A}^{[0]} \tag{S3}$$
$$\mathbf{Z}^{[l]} = \mathbf{W}^{[l]} * \mathbf{A}^{[l-1]} + b^{[l]} \tag{S4}$$
$$\mathbf{A}^{[l]} = \mathrm{ReLu}\left(\mathbf{Z}^{[l]}\right) \tag{S5}$$

The above equations describe linear-nonlinear transformations of the input as its passed through sequential layers. For all layers l, $*$ is the discrete 2D convolution operator. Values for $W^{[l]}$ and $b^{[l]}$ constitute filters in an F-dimensional space, and are learned by training on the ImageNet1K database[3] for object recognition (not segmentation).

For each layer $[l]$ we use a linear transformation, principal component analysis (PCA), to reduce the dimensionality of the observations[6] to M. We set $M = 6$ to standardize feature dimensionality across layers, and because it typically explained 95% of the variance in the shallowest layers. The projection onto principal components is a simple matrix multiplication:

$$\mathbf{Z}^{[l]}\mathbf{\Phi}^{[l]} = \mathbf{X}^{[l]} \tag{S6}$$
$$\mathbf{X}^{[l]} \in \mathbb{R}^{h\times w\times 6} \tag{S7}$$

where principal components are the columns of $\mathbf{\Phi}^{[l]}$. Therefore, $\mathbf{X}^{[l]}$ is a 6-dimensional representation of the features at layer l in image data.

As in the original FlexMM implementation, to improve segmentation performance, we concatenated the low-dimensional representation at any given layer to the PCA projection of RGB features $\mathbf{X}^{[0]}$. We then defined the feature vector at layer l:

$$\vec{x}_i^{[l]} = \left[\mathbf{X}_i^{[0]}, \mathbf{X}_i^{[l]}\right] \tag{S8}$$

A.1. Feature selection. For each image, we selected, out of the first five convolutional layers, the layer whose features led to model segmentation maps most similar to the participant's segmentation maps decoded from the task (with similarity measured using the adjusted Rand Index[1]). Therefore the layer of VGG-19 used was tuned to each image for each participant.

We found that intermediate layers of VGG-19 led to results best aligned with human RT-distance correlations. When features were selected from layers deeper than layer 5 or from only layer 1, model correlations were weaker. It is thought that early layers of VGG-19 have access to bottom-up image feature information such as edges, texture, color, and luminance[3]. Furthermore, because VGG-19 is a network trained for object recognition with backpropagation, deeper layers should have more access to object–specific or semantic information. Therefore, our feature basis partially accounts for semantic or object priors in human participants, but is not fully abstracted away from lower–level image information.

bioRxiv preprint doi: https://doi.org/10.64898/2026.01.30.702842; this version posted February 2, 2026. The copyright holder for this preprint (which was not certified by peer review) is the author/funder, who has granted bioRxiv a license to display the preprint in perpetuity. It is made available under aCC-BY 4.0 International license.

B. Gaussian scale mixtures. To simplify our notation we remove the layer–indexing from here on. Bold, italic letters indicate a vector random variable at a single pixel i ($\vec{x}_i \equiv \boldsymbol{x}$). Let $\boldsymbol{x} \in \mathbb{R}^{12}$ be the random variable representation of a single (*i.e.* at one pixel) multidimensional feature vector.

We can define the prior for $\boldsymbol{x}$ rigorously through normative theories of natural vision. It has been shown that natural images present with the statistics of a Gaussian Scale Mixture (GSM), which is a standard multivariate normal $\boldsymbol{g} \in \mathbb{R}^{12}$ multiplied by a random global scalar $v \in \mathbb{R}^+$. The components of the multivariate normal represent the weight with which local features (such as edges with a specific orientation and spatial frequency) contribute to the input image, while the scalar multiplier represents a global modulator such as contrast [7–11]. In our formulation, we use the Student-t distribution, which is rewritten as a Gaussian Scale Mixture by scaling the covariance of a multidimensional Gaussian, $\boldsymbol{\Sigma}$, using a scalar v, that is itself a random variable sampled from a one–dimensional inverse Chi-square distribution scaled by its degrees of freedom ν. Ultimately we write:

$$\begin{aligned}\boldsymbol{x} &= v\boldsymbol{g} + \vec{\mu}\\ v^2 &\sim \nu \cdot \text{Inv-}\chi^2(\nu)\\ \boldsymbol{g} &\sim \mathcal{N}(0, \boldsymbol{\Sigma})\\ \boldsymbol{x} &\sim \mathcal{N}(\vec{\mu}, v^2 \cdot \boldsymbol{\Sigma})\end{aligned}$$

Compute the PDF of $\boldsymbol{x}$, $p(\boldsymbol{x})$ by marginalizing over v

$$p(\boldsymbol{x}) = \int_v p(\boldsymbol{x}|v)p(v)dv \tag{S9}$$

$$= \int_v \frac{1}{(2\pi)^6 \left|v^2\boldsymbol{\Sigma}\right|^{\frac{1}{2}}} \exp\left(\frac{(\boldsymbol{x}-\vec{\mu})^\top \boldsymbol{\Sigma}^{-1}(\boldsymbol{x}-\vec{\mu})}{-2v^2}\right) p(v)dv \tag{S10}$$

The integral in equation S10 can be written as the expression f_1 [12]:

$$f_1(\boldsymbol{x}, \nu, \vec{\mu}, \boldsymbol{\Sigma}) = \frac{\Gamma\left(\frac{\nu+12}{2}\right)|\boldsymbol{\Sigma}|^{-\frac{1}{2}}}{(\pi\nu)^6 \Gamma\left(\frac{\nu}{2}\right)\left(1 + (\boldsymbol{x}-\vec{\mu})^\top \boldsymbol{\Sigma}^{-1}(\boldsymbol{x}-\vec{\mu})/\nu\right)^{\frac{\nu+12}{2}}} \tag{S11}$$

Where Γ is the gamma function. This formulation of the Student distribution makes our parametrization explicit, $\boldsymbol{\theta} = (\nu, \vec{\mu}, \boldsymbol{\Sigma})$.

B.1. Mixtures of Gaussian Scale Mixtures. The objective now is to learn a *mixture* of GSMs such that each vector $\boldsymbol{x}$ may be assigned to one of K classes (classes in our formulation are segments). Classes are notated as one-hot encoded vectors and we write $p(\boldsymbol{c})$ refer to the corresponding probability, *e.g.* if there were 3 classes, class 2 would be represented as: $[0,1,0]$ with corresponding probability $p(c_2) \equiv p(\boldsymbol{c} = [0,1,0])$.

Classes are assigned with a prior probability vector denoted $\boldsymbol{\pi}$ which is an element of the $K-$dimensional simplex $\boldsymbol{\pi} \in \Delta^K$, namely all its elements must sum to one. The PDF of $\boldsymbol{x}$ is now simply a weighted sum:

$$p(\boldsymbol{x}) = \sum_{k=1}^{K} \pi_k f_1(\boldsymbol{\theta}_k) \tag{S12}$$

Where π_k is the kth-element of the vector $\boldsymbol{\pi}$, and $\boldsymbol{\theta}_k$ is the set of parameters $(\nu_k, \vec{\mu}_k, \boldsymbol{\Sigma}_k)$ that is unique to the k-th class.

C. Defining the graphical model for FlexMM. In Supp. Fig. 11, we define our mixture of GSMs as a graphical model, specifically a Bayesian Network, where for two random variables $\boldsymbol{r}_1$ and $\boldsymbol{r}_2$, the notation $\boldsymbol{r}_1 \rightarrow \boldsymbol{r}_2$ indicates a conditional relationship $p(\boldsymbol{r}_2 \mid \boldsymbol{r}_1)$ [13]. Following the FlexMM family of probabilistic mixture models [6], to introduce the spatial prior, we add extra edges to the graph (Supp. Fig. 11, blue lines). These edges ultimately define a latent variable $\boldsymbol{B} \in \mathbb{R}^K$, the concentration parameter of the Dirichlet distribution of $\boldsymbol{\pi}$. Only one $\boldsymbol{B}$ node is shown in Supp. Fig. 11 for the central pixel in a 3×3 neighborhood of pixels. However, there is a $\boldsymbol{B}$ node for every pixel which is influenced by that pixel's neighbors.

The extra edges inserted into the model cause a loop. Learning and inference can be performed despite the presence of the loop, as we will discuss in the next section.

bioRxiv preprint doi: https://doi.org/10.64898/2026.01.30.702842; this version posted February 2, 2026. The copyright holder for this preprint (which was not certified by peer review) is the author/funder, who has granted bioRxiv a license to display the preprint in perpetuity. It is made available under aCC-BY 4.0 International license.

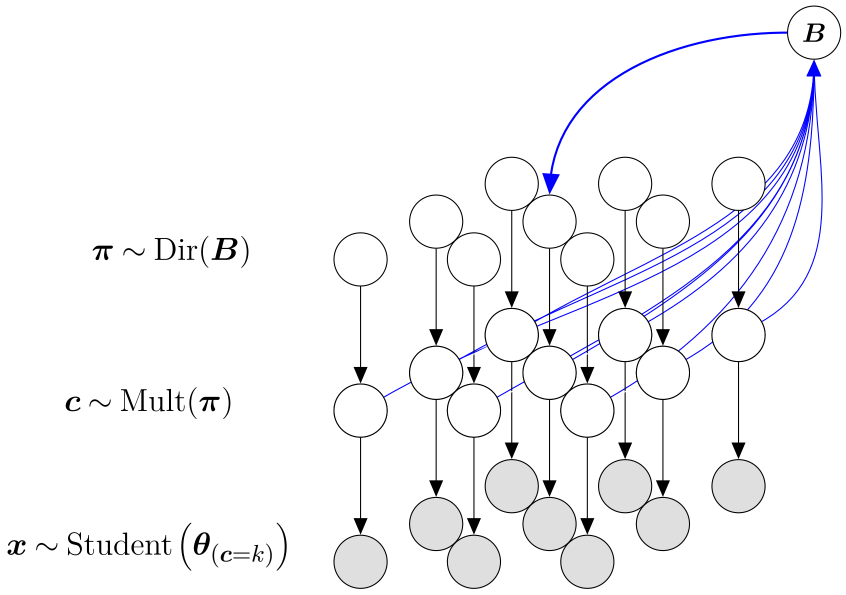

Supplementary Figure 11: A probabilistic graphical model for a 3 × 3 pixel input. We define observed random variables as $\boldsymbol{x}$, these variables are distributed according to a Student distribution with parameters $\{\boldsymbol{\theta}\}_{\boldsymbol{c}=k}$. The parameters are determined based on feature classes $\boldsymbol{c}$. These prior probabilities of these class labels are defined by the random variable $\boldsymbol{\pi}$, distributed according to a Dirichlet distribution. A neighborhood (which is 3 × 3 pixels, in this illustration), of surrounding class labels defines the hyperparameter $\boldsymbol{B}$ (only one $\boldsymbol{B}$ is shown) which is the concentration parameter of the Dirichlet distribution. Defining $\boldsymbol{B}$ on the basis of the class labels of surrounding pixels ensures that $\boldsymbol{\pi}$ variables that are spatially close to each other are more likely to be similar.

D. Learning and inference dynamics. The main goal of this section is to provide a derivation of equations 1,2 of the main text, which our results are based on. As with any probabilistic graphical model, there are multiple algorithms that can be considered for learning and inference[13]. For FlexMM[6] we use a modified version of the Expectation-Maximization algorithm[14] that guarantees closed-form linear updates for the class probabilities and flexibility in how class information is integrated across pixels to define the $\boldsymbol{B}$ variables. The implementation and proof of convergence for FlexMM can be found in our earlier work[6].

In the following sections D.1 and D.2, we focus on alternative mathematical views of our model's dynamics in two ways, to highlight links to normative theories of perceptual dynamics. First, we briefly mention the recurrent message-passing dynamics that are present throughout the lattice of pixels to connect our work to normative theories of recurrence. Second, we use a view of EM as variational approximate inference to show that iteration is used to minimize statistical free-energy, which leads to a simplified derivation of the update rule in equations 1,2.

We note that disentangling learning (*i.e.* estimating parameters given data) from inference (*i.e.* forming the posterior distribution, given the data and parameters) requires some care for our graphical model. FlexMM alternates between an M step in which it learns parameters, including a prior probability for the class labels, and an E step in which it infers the posterior class probabilities given those parameters. In the model with the spatial prior (e.g. Fig. 3a, also Supp. Fig. 11), the parameters $\boldsymbol{\pi}$ are learned per-pixel, so the inferred posterior probability is proportional to the prior. In models without a spatial prior (e.g. Fig. 5f), a single prior probability for all pixels is learned during the M-step, and inferring a posterior probability over segments means that only the E-step generates a probabilistic segmentation map. We will discuss this distinction in greater detail in Section E when discussing the reduced versions of IBI.

In all versions of IBI and Expectation-Maximization in general, the prior is iteratively updated to generate a posterior and vice–versa. Therefore, technically speaking, the first prior our model learns is based on the initial guess which comes from participants segmentation maps. This initial guess introduces information about the segments perceived by the participants, which are likely based on both bottom-up features as well as object and scene understanding.

D.1. EM as recurrent message passing. The Bayesian Network (Supp. Fig. 11) can be rewritten per-pixel with constraints on the relationships between variables. This formalization is called a factor graph[13]. Supplementary Figure 12 is a per-pixel version of Fig. 3a and Fig. 11 formalized as a factor graph. The factor graph depicts informational relationships with undirected edges, random variable nodes $\mathcal{V}$ and "factor nodes", $\mathcal{F}$. Factor nodes $\mathcal{F}$ elucidate the nature of the relationships between variables using the definitions of selected distributions. The joint probability distribution of the nodes $\mathcal{V}$ is then written as the product of factors: $p(\mathcal{V}) = \frac{1}{Z}\prod_{a\in\mathcal{F}} f_a$, where Z is a normalization constant. Specifically, we use the following factorization:

bioRxiv preprint doi: https://doi.org/10.64898/2026.01.30.702842; this version posted February 2, 2026. The copyright holder for this preprint (which was not certified by peer review) is the author/funder, who has granted bioRxiv a license to display the preprint in perpetuity. It is made available under aCC-BY 4.0 International license.

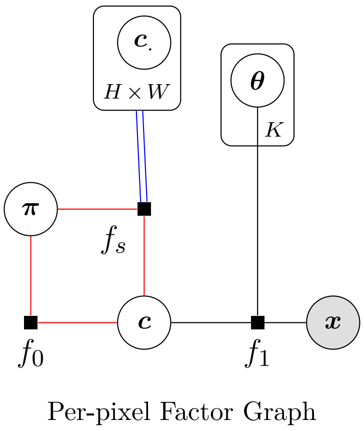

Supplementary Figure 12: A factor graph of the probabilistic dependencies influencing each pixel. Compared to the probabilistic graphical model of Supp. Fig. 11, directed edges have been replaced with undirected edges and factor nodes. Rounded rectangles around nodes are referred to as "plates" and the annotation within the plate indicates how many values of that random variable are present. Specifically, there are $H \times W$ neighboring class labels used in the per-pixel class and K sets of parameters. The red lines indicate a loop in the graph. To simplify, the random variable $\boldsymbol{B}$ is not shown as a node, but is implicitly accounted for through factor f_s (eq. S15). The double blue lines indicate that information from multiple neighbors in used in the spatial prior *i.e.* $\boldsymbol{c}$ values over a local set of $H \times W$ pixels regularize the value of $\boldsymbol{\pi}$. f_0, f_1, and f_s are factors of the joint probability distribution *i.e.* $p(\boldsymbol{x}, \boldsymbol{\pi}, \boldsymbol{c}, \boldsymbol{c}_{H\times W}, \boldsymbol{B}, \boldsymbol{\theta}) = \frac{1}{Z}(f_s \cdot f_0 \cdot f_1)$.

$$f_0(\boldsymbol{\pi}, \boldsymbol{c}) = p(\boldsymbol{c}|\boldsymbol{\pi}) = \text{Mult}(\boldsymbol{\pi}) \quad \textbf{(S13)}$$
$$f_1(\boldsymbol{c}, \boldsymbol{x}, \boldsymbol{\theta}) = p(\boldsymbol{x}|\boldsymbol{c}, \boldsymbol{\theta}) = \text{Student}(\boldsymbol{\theta}) \quad \textbf{(S14)}$$
$$f_s(\boldsymbol{\pi}, \boldsymbol{c}, \boldsymbol{c}_0, ..., \boldsymbol{c}_{H\times W}) = p(\boldsymbol{\pi}|\boldsymbol{B})p(\boldsymbol{B}|\boldsymbol{c}_0, ..., \boldsymbol{c}_{H\times W}) \quad \textbf{(S15)}$$

Equation S15 describes how a spatial prior of size $H \times W$ (where $H \leq h$ and $W \leq w$, the full image size) is implemented where $\boldsymbol{B}$ is a Dirichlet parameter vector that has the same dimensionality as $\boldsymbol{\pi}$[15], therefore:

$$p(\boldsymbol{\pi}|\boldsymbol{B}) = \text{Dirichlet}(\boldsymbol{B}) \quad \textbf{(S16)}$$
$$p(B_k|\boldsymbol{c}_0, ..., \boldsymbol{c}_{H\times W}) = \delta(u(c_{0,k}, ..., c_{H\times W,k})) \quad \textbf{(S17)}$$

where $u : \mathbb{R}^{H\times W\times K} \rightarrow \mathbb{R}^K$ can be any linear function, and δ is the Dirac delta function.

Our factor graph is written with a loop (Fig. 12), but previous work using the EM algorithm on loopy factor graphs[16], has shown that the Expectation and Maximization steps can be considered as message-passing along two separate "tree–like" graphs, and that if neither of them contains loops the EM algorithm can be implemented exactly (see Dauwels[17] for the explicit form of messages used in EM). This is the case for our model[6] (Supp. Fig. 13).

One advantage of describing FlexMM in terms of message-passing is that it highlights the connection of FlexMM to normative and mechanistic theories that used this powerful computational scheme to clarify the role of recurrence (both lateral recurrence and feedback connections) in probabilistic neural computations[18–20].

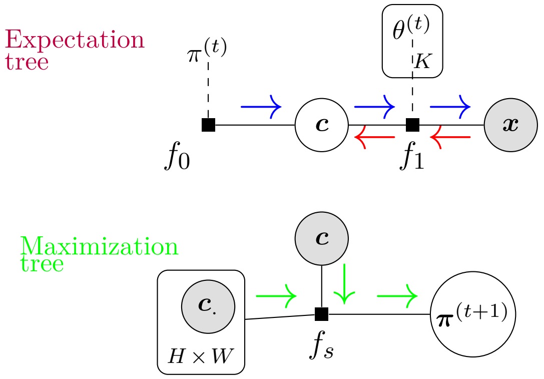

Supplementary Figure 13: Breaking the loopy model using EM. The Expectation tree (top row) and the Maximization tree (bottom row) generated by "unrolling" the loopy model. Arrows represent messages passed along the edges. $\pi^{(t)}$ and $\theta^{(t)}$ are known values of parameters at the current time step and therefore not written as variable nodes here. The algorithm for computing the expectation in the Expectation Step is a forward-backward sum-product message-passing algorithm[17]. The blue arrows show the forward pass while the red arrows show the backward pass. In the Maximization tree, the spatial prior is implemented using max-product message passing with messages represented by green arrows[17].

bioRxiv preprint doi: https://doi.org/10.64898/2026.01.30.702842; this version posted February 2, 2026. The copyright holder for this preprint (which was not certified by peer review) is the author/funder, who has granted bioRxiv a license to display the preprint in perpetuity. It is made available under aCC-BY 4.0 International license.

D.2. EM as variational inference. Having discussed the view of EM as a message–passing algorithm that connects to theories of recurrence, we now present an alternative view of EM iterations as approximate variational inference[15,21–23]. This view emphasizes that iteration in EM is a form of minimizing statistical free-energy, and can in principle, be seen as gradient descent where information from the prior helps determine the direction of optimal descent[23]. We will use this view to present an abridged derivation.

Implementing the EM algorithm as variational inference involves treating $\boldsymbol{\pi}$ and $\boldsymbol{\theta}$ as parameters in order to learn the marginal distribution $p(\boldsymbol{x}|\boldsymbol{\pi},\boldsymbol{\theta})$ that best explains the observations $\boldsymbol{x}$ at all pixels. Here, we will not explicitly discuss the role of the Dirichlet as a spatial prior, but focus on dynamics. For a set of observations $\mathbf{X}$ over all $h \times w$ pixels, we begin with the assumption that a single observation $\boldsymbol{x}_i$ is independent from others. In this section, the spatial index i is explicitly written.

The log-probability of the observations factorizes as follows:

$$\log p(\mathbf{X}|\boldsymbol{\Pi},\boldsymbol{\Theta}) = \sum_{i}^{h\times w} \log p(\boldsymbol{x}_i \mid \boldsymbol{\pi}_i, \boldsymbol{\theta}) \tag{S18}$$

We now introduce the latent variable $\boldsymbol{c}_i$, a class label, for each observation at pixel i. As in earlier sections, classes are notated as one-hot encoded vectors, *e.g.* if there were 3 classes, class 2 would be represented as: [0, 1, 0] with corresponding probability $p(c_{i,2}) \equiv p(\boldsymbol{c}_i = [0,1,0])$. Then:

$$\log p(\mathbf{X}|\boldsymbol{\Pi},\boldsymbol{\Theta}) = \sum_{i}^{h\times w} \log \sum_{k}^{K} p\left(\boldsymbol{x}_i, c_{i,k} \mid \pi_{i,k}, \boldsymbol{\theta}_k\right) \tag{S19}$$

Equation S19 defines the observation likelihood as the marginal likelihood over $\boldsymbol{c}_i$. To move the summation outside the logarithm we can introduce an approximate distribution for $\boldsymbol{c}_i$, namely $\boldsymbol{c}_i \sim q^{(t)}$.

$$= \sum_{i}^{h\times w} \log \sum_{k}^{K} \frac{q^{(t)} \cdot p\left(\boldsymbol{x}_i, c_{i,k} \mid \pi_{i,k}, \boldsymbol{\theta}_k\right)}{q^{(t)}} \tag{S20}$$

The above can be rewritten using the expectation operator $\mathbb{E}_{\boldsymbol{c}_i \sim q^{(t)}}$, which is the expectation with respect to the approximate distribution $q^{(t)}$. Using the expectation operator:

$$= \sum_{i}^{h\times w} \log \left(\mathbb{E}_{\boldsymbol{c}_i \sim q^{(t)}} \left[\frac{p(\boldsymbol{x}_i, \boldsymbol{c}_i \mid \boldsymbol{\pi}_i, \boldsymbol{\theta})}{q^{(t)}} \right] \right) \tag{S21}$$

$$= \sum_{i}^{h\times w} \log \left(\mathbb{E}_{\boldsymbol{c}_i \sim q^{(t)}} \left[\frac{p\left(\boldsymbol{c}_i \mid \boldsymbol{x}_i, \boldsymbol{\pi}_i\right) \cdot p\left([\boldsymbol{x}_i \mid \boldsymbol{\theta}\right)}{q^{(t)}} \right] \right) \tag{S22}$$

Using Jensen's inequality we can move the logarithm inside the summation, and establish a lower bound to maximize.

$$\geq \sum_{i}^{h\times w} \mathbb{E}_{\boldsymbol{c}_i \sim q^{(t)}} \left[\log p(\boldsymbol{c}_i \mid \boldsymbol{x}_i, \boldsymbol{\pi}_i)\right] + \mathbb{E}_{\boldsymbol{c}_i \sim q^{(t)}} \left[\log p\left(\boldsymbol{x}_i \mid \boldsymbol{\theta}\right)\right] - \mathbb{E}_{\boldsymbol{c}_i \sim q^{(t)}} \left[\log q^{(t)}\right] \tag{S23}$$

Inference aims to find the approximate distribution q to maximize the above lower-bound monotonically. This is functionally equivalent to minimizing the negative of this quantity, which can be interpreted as a quantity called variational free energy[21,22,24] (note that the third term is the statistical entropy of the approximate distribution $q^{(t)}$).

Note that the challenge with performing inference with the model as written in equation S24 is that in the first term:

$$\sum_{i}^{h\times w} \mathbb{E}_{\boldsymbol{c}_i \sim q^{(t)}} \left[\log p(\boldsymbol{c}_i \mid \boldsymbol{x}_i, \boldsymbol{\pi}_i)\right] \tag{S24}$$

there are $(K-1) \cdot h \cdot w$ parameters and only $h \cdot w$ observations. Therefore, to effectively reduce the number of parameters we use regularization: we introduce a linear function u that can be applied over the $\boldsymbol{\pi}_i$ within a neighborhood of size $H \times W$ to facilitate spatial smoothing. In practice, special care must be taken to define the u as linear using additional parameters from conjugate priors (see previous Section D.1 and the FlexMM paper[6], particularly

bioRxiv preprint doi: https://doi.org/10.64898/2026.01.30.702842; this version posted February 2, 2026. The copyright holder for this preprint (which was not certified by peer review) is the author/funder, who has granted bioRxiv a license to display the preprint in perpetuity. It is made available under aCC-BY 4.0 International license.

Appendix B for proofs). We ignore these additional parameters in the current derivation to focus on one main point — the u function must be linear as applied to the expectation so that it can be taken out of the expectation term by the linearity of expectation:

$$\mathbb{E}_{\boldsymbol{c}_i \sim q^{(t)}}\left[u\left(\log p(c_{\cdot,k} \mid x_{\cdot}, \pi_{\cdot,k})\right)\right] = u_{i,k}\left(\mathbb{E}_{\boldsymbol{c}_{\cdot} \sim q^{(t)}}\left[\log p(c_{\cdot,k} \mid x_{\cdot}, \pi_{\cdot,k})\right]\right) \tag{S25}$$

To apply the condition that closer pixels are more likely to be in the same class we use as the u function a convolution with a discrete 2D Gaussian with finite kernel size.

D.3. Per-pixel EM. Notice that the linear u function can be applied to the standard formulation of the expected complete-log-likelihood over neighbors. The standard formulation of EM states that parameters evolve to increase the likelihood of the dataset as a batch[14]. In our case, parameters are per-pixel. Examining the likelihood traces in our model we found that certain pairs of pixels reach their final likelihood value before other pairs. Therefore it is worth a brief look at existing theory on EM with single observations, termed incremental EM, which was shown to be feasible through the variational view of EM[21]. Neal and Hinton have shown that maximizing $\log p(\mathbf{X} \mid \mathbf{\Pi}, \boldsymbol{\theta})$ is equivalent to maximizing the complete log-likelihood per-pixel when latent observations $\{\boldsymbol{c}\}_{\forall i}$ are independent. In our formulation strictly speaking, $\{\boldsymbol{c}\}_{\forall i}$ are not independent because of the u function. However, because $\boldsymbol{\pi}_i$ are also learned per pixel we can treat $\{\boldsymbol{c}\}_{\forall i}$ as conditionally independent when $\boldsymbol{\pi}_i$ is known for all i. This justifies that single pixel updates are unique while increasing likelihood for the whole image.

D.4. EM as gradient ascent. Having established that we can apply u to the standard complete-log-likelihood, and that this quantity can be optimized per pixel, we can introduce a per-pixel objective functional $\mathcal{O}_i$ by defining a parameter space $\{\boldsymbol{\pi}_i, \boldsymbol{\theta}\} \in \vartheta$ and a distributional space $q \in \mathcal{Q}$. The way that iteration enables gradient ascent for this functional is summarized in Table 1, and aligns with normative work exploring dynamic sampling circuits[25–27]. The equation for the per-pixel objective functional at time step (t) (ignoring the entropy contribution from equation S23) is:

$$\mathcal{O}_i(\mathcal{Q}, \boldsymbol{\vartheta}) = \mathbb{E}_{\boldsymbol{c}_i \sim q^{(t)}}\left[\log p(\boldsymbol{x}_i, \boldsymbol{c}_i \mid \boldsymbol{\pi}_i, \boldsymbol{\theta})\right] \tag{S26}$$

Consider the derivative $\frac{\partial \mathcal{O}_i}{\partial \mathcal{Q}}$. Past work has shown[21] that when setting $\frac{\partial \mathcal{O}_i}{\partial \mathcal{Q}} = 0$ a maximum exists at: $q^{\dagger} = p(\boldsymbol{c}|\boldsymbol{x})$. At any given iteration t, computing $q^{\dagger(t)} = p(\boldsymbol{c}|\boldsymbol{x})$ is the expectation step, while the maximization step involves learning parameters $\left(\boldsymbol{\pi}_i^{(t)}, \boldsymbol{\theta}^{(t)}\right)$ to further improve approximation. The EM update rule as derived below uses parametrized distributions to make these computations exactly, but it is nevertheless a specialized form of gradient-based optimization with the goal of approximation. These closed–form expressions meaningfully capture dynamics because constrained optimization (*i.e.* $\boldsymbol{\pi}$ must sum up to one) require a small step size. This was shown by Xu and Jordan[28] who explicitly relate EM to iterative Newton and Quasi-Newton's methods.

To continue the derivation, for the M step we re-express the expectation in equation S26 explicitly as a sum over class labels and we introduce explicitly the time labels:

$$\mathcal{O}_i\left(q^{\dagger(t)}, \boldsymbol{\vartheta}\right) = \sum_{k}^{K} \left(\log p\left(\boldsymbol{x}_i, c_{i,k} \,\middle|\, \pi_{i,k}, \boldsymbol{\theta}_k\right)\right) \cdot p\left(c_{i,k} | \boldsymbol{x}_i, \pi_{i,k}^{(t-1)}, \boldsymbol{\theta}_k^{(t-1)}\right) \tag{S27}$$

We use Bayes' rule for the conditional probability term:

$$p\left(c_{i,k} \,\middle|\, \boldsymbol{x}_i, \pi_{i,k}^{(t-1)}, \boldsymbol{\theta}_k^{(t-1)}\right) = \frac{p\left(\boldsymbol{x}_i \,\middle|\, c_{i,k}, \boldsymbol{\theta}_k^{(t-1)}\right) p\left(c_{i,k} \,\middle|\, \pi_{i,k}^{(t-1)}\right)}{p(\boldsymbol{x}_i)} \tag{S28}$$

	Recurrent step	Gradient step
(E-step)	Hold $\pi_{i,k}^{(t-1)}$ and $\boldsymbol{\theta}_k^{(t-1)}$ constant for all i	$\frac{\partial \mathcal{O}_i}{\partial \mathcal{Q}}$; find local maximum at $q^{\dagger(t)}$
(M-step)	Hold $q^{\dagger(t)}$ constant	$\frac{\partial \mathcal{O}_i}{\partial \boldsymbol{\vartheta}}$; find local maximum

Table 1: EM framed as gradient ascent[21,23,28]. This framing shows how different pixels each contribute to increasing the overall data likelihood as long as there is information in $q^{\dagger}$ about neighbors. The update rule in equation 2 is derived by finding closed-form solutions for local maxima when setting the gradient steps equal to 0 and then applying the function u. Values for $\pi_{i,k}^{(t)}$ are updated until the total likelihood $\log p(\mathbf{X} \mid \mathbf{\Pi}, \boldsymbol{\theta})$ reaches an asymptote.

bioRxiv preprint doi: https://doi.org/10.64898/2026.01.30.702842; this version posted February 2, 2026. The copyright holder for this preprint (which was not certified by peer review) is the author/funder, who has granted bioRxiv a license to display the preprint in perpetuity. It is made available under aCC-BY 4.0 International license.

We write the denominator as the marginalized sum over all classes. We also introduce the symbol $\gamma_{i,k}^{(t)}$, termed the "responsibility" which quantifies how "responsible" a particular class is for a particular observation.

$$\gamma_{i,k}^{(t)} \equiv \frac{p\left(\boldsymbol{x}_i \middle| c_{i,k}, \boldsymbol{\theta}_k^{(t-1)}\right) p\left(c_{i,k} \middle| \pi_{i,k}^{(t-1)}\right)}{\sum_k^K p\left(\boldsymbol{x}_i \middle| c_{i,k}, \boldsymbol{\theta}_k^{(t-1)}\right) p\left(c_{i,k} \middle| \pi_{i,k}^{(t-1)}\right)} \tag{S29}$$

Now we can substitute equation S29 into equation S27:

$$\mathcal{O}_i\left(q^{\dagger(t)}, \boldsymbol{\vartheta}\right) = \sum_k^K \left(\log p\left(\boldsymbol{x}_i, c_{i,k} \,\middle|\, \pi_{i,k}, \boldsymbol{\theta}_k\right)\right) \cdot \frac{p\left(\boldsymbol{x}_i \middle| c_{i,k}, \boldsymbol{\theta}_k^{(t-1)}\right) p\left(c_{i,k} \middle| \pi_{i,k}^{(t-1)}\right)}{\sum_k^K p\left(\boldsymbol{x}_i \middle| c_{i,k}, \boldsymbol{\theta}_k^{(t-1)}\right) p\left(c_{i,k} \middle| \pi_{i,k}^{(t-1)}\right)} \tag{S30}$$

$$= \sum_k^K \left(\log p\left(\boldsymbol{x}_i, c_{i,k} \,\middle|\, \pi_{i,k}, \boldsymbol{\theta}_k\right)\right) \cdot \gamma_{i,k}^{(t)} \tag{S31}$$

Decompose the joint probability distribution

$$= \sum_k^K \left(\log\left(p\left(\boldsymbol{x}_i \,\middle|\, c_{i,k}, \boldsymbol{\theta}_k\right) \cdot p\left(c_{i,k} \middle| \pi_{i,k}\right)\right)\right) \cdot \gamma_{i,k}^{(t)} \tag{S32}$$

$$= \sum_k^K \left[\log\left(\text{Student}\left(\boldsymbol{\theta}_k\right)\right) + \log\left(\pi_{i,k}\right)\right] \cdot \gamma_{i,k}^{(t)} \tag{S33}$$

Here we show the maximization with respect to $\pi_{i,k}$ (see Peel[12] for estimation of the parameters $\boldsymbol{\theta}_k$). Maximization for $\pi_{i,k}$ is subject to the constraint $\sum_k^K \pi_{i,k} = 1$, implemented with a Lagrange multiplier λ:

$$\mathcal{O}_i\left(q^{\dagger(t)}, \pi_{i,k}, \lambda\right) = \gamma_{i,k}^{(t)} \cdot \log\left(\pi_{i,k}\right) + \lambda\left(1 - \sum_k^K \pi_{i,k}\right) \tag{S34}$$

$$\frac{\partial \mathcal{O}_i}{\partial \pi_{i,k}} = \frac{\gamma_{i,k}^{(t)}}{\pi_{i,k}} - \lambda \tag{S35}$$

Set the partial derivative equal to 0 and solve for $\pi_{i,k}$ to find the updated value $\pi_{i,k}^{(t)}$:

$$\lambda = \frac{\gamma_{i,k}^{(t)}}{\pi_{i,k}} \tag{S36}$$

$$\pi_{i,k}^{(t)} = \frac{\gamma_{i,k}^{(t)}}{\lambda} \tag{S37}$$

Equation S37 is the standard formulation of the EM update rule, because $\sum_k^K \pi_{i,k}^{(t)} = 1$ we can write that $\lambda = \sum_k^K \gamma_{i,k}^{(t)}$. Our formulation differs however because of the u function applied to the complete log-likelihood so we write:

$$\lambda = \frac{u_i\left(\gamma_{\cdot,k}^{(t)}\right)}{\pi_{i,k}} \tag{S38}$$

Finally, this leads to our update rule:

$$\boxed{\pi_{i,k}^{(t)} = \frac{u_i\left(\gamma_{\cdot,k}^{(t)}\right)}{\sum_k^K u_i\left(\gamma_{\cdot,k}^{(t)}\right)}} \tag{S39}$$

Xu and Jordan[28] have shown that EM can be considered a quasi–Newton's method, which is a classical iterative optimization method.

We end with a summary of how our approximate distribution q changes with time. We started with an approximate distribution over $\boldsymbol{c}$, $\boldsymbol{c} \sim q$. We show that to maximize likelihood/minimize free-energy $q \to q^\dagger \equiv p(\boldsymbol{c}|\boldsymbol{x}) \equiv \gamma^{(t)}$ which is then rescaled to $\pi^{(t)}$ and that through spatial smoothing with u, $\boldsymbol{\pi}$ has further, single-pixel dynamics π_i.

bioRxiv preprint doi: https://doi.org/10.64898/2026.01.30.702842; this version posted February 2, 2026. The copyright holder for this preprint (which was not certified by peer review) is the author/funder, who has granted bioRxiv a license to display the preprint in perpetuity. It is made available under aCC-BY 4.0 International license.

D.5. Deep spatial prior. As explained in Section A, we use the notation $\boldsymbol{x}^{[l]}$ to indicate that our random variable formulation of the observations $\mathbf{X}$ is in a subspace of $\mathbf{Z}^{[l]}$ for a specific layer $[l]$. This leads to latent variables being layer specific as well (e.g. $\boldsymbol{c}^{[l]}$, which was omitted in earlier sections for clarity). Just as class labels $\boldsymbol{c}$ that are close together in space are more likely to be the same, we assumed that so too should class labels for the same location across neighboring layers $\left\{\boldsymbol{c}^{[l-1]}, \boldsymbol{c}^{[l]}, \boldsymbol{c}^{[l+1]}\right\}$. This leads to better segmentation performance with FlexMM[6].

Therefore, we can write equation S24 for a spatial neighborhood while considering neighboring layers as well:

$$\pi_{i,k}^{(t)[l]} = \frac{u_i\left(\gamma_{\cdot,k}^{(t)[l-1]}, \dots, \gamma_{\cdot,k}^{(t)[l+1]}\right)}{\sum_k^K u_i\left(\gamma_{\cdot,k}^{(t)[l-1]}, \dots, \gamma_{\cdot,k}^{(t)[l+1]}\right)} \tag{S40}$$

The appropriate linear function u for applying this deep spatial prior and the corresponding update rule is presented in Box I of our FlexMM paper[6].

D.6. EM initial guess. For our initial guesses, $\pi_{i,k}^{(t=0)}$ and $\theta_k^{(t=0)}$, first we define $\pi_{i,k}^{(0)}$ by sampling from resized, probabilistic segmentation maps of the participants (Fig. 1b, top). (Resizing is necessary because the coordinate system for participant segmentation maps is lower resolution than the per-pixel coordinate system of FlexMM). Let the value at each pixel in this resized participant segmentation map be notated $\boldsymbol{p}$ (analogous to $\boldsymbol{\pi}$ from FlexMM, that we have discussed in detail, but based on participant responses). We can then define sampled labels:

$$s_i \sim \mathrm{Mult}(\boldsymbol{p}) \tag{S41}$$

We then compute $\pi_{i,k}^{(t=0)}$ from s_i like so.

$$\pi_{i,k}^{(t=0)} = \begin{cases} 1-\epsilon & \text{if} \quad k = s_i \\ 0+\epsilon/(K-1) & \text{if} \quad k \neq s_i \end{cases} \tag{S42}$$

After computing this mapping, we estimate $\mu_k^{(0)}$ and $\mathbf{\Sigma}_k$ using assignments s_i. For the initial guess of ν we use $\nu = 2$, which simplifies the mixer distribution $\mathbb{P}(v)$ to a scaled inverse Rayleigh distribution[6,11].

Note that by sampling in the initial guess, our model is sensitive to different levels of perceptual uncertainty in each case (*i.e.* each participant and image). This is part of the reason we refer to the EM initial guess as the participant's mental map in the main text. While the final maps from FlexMM are different depending on the precise sample of initial guess, our main findings on dynamics are qualitatively robust across varied initial guesses.

E. Graphical model perturbations.

In this section we focus on the ablations detailed in Figure 5.

E.1. No local connectivity model. If we define the u function as a Gaussian with an infinitely wide kernel *i.e.* $u(\gamma_{N,k}^{(t)}) = \sum_{i=1}^N \gamma_{i,k}^{(t)}$, where $N = h \times w$, then we can plug back into equation S29 to get:

$$\pi_{i,k}^{(t)} = \frac{\sum_{i=1}^N \gamma_{i,k}^{(t)}}{\sum_{i=1}^N \sum_{k=1}^K \gamma_{i,k}^{(t)}} \tag{S43}$$

By definition, $\sum_{i=1}^N \sum_{k=1}^K \gamma_{i,k}^{(t)} = N$ so:

$$\pi_k^{(t)} = \frac{\sum_{i=1}^N \gamma_{i,k}^{(t)}}{N} \tag{S44}$$

Because we are summing over all i, the index i is no longer present on the left-hand-side of equation S44. Therefore, to obtain per-pixel probabilities, we have to compute the posterior (which is also equivalent to the E step of the next iteration):

$$q_{i,k}^{(t)} = p\left(\boldsymbol{c} = k \mid \pi_k^{(t)}, \theta_k^{(t)}\right) \tag{S45}$$

<u>Further Discussion:</u>

Because the no local connectivity model yielded similar performance to the full IBI model (Fig. 5), we take a closer look at the nature of the no local connectivity perturbation.

bioRxiv preprint doi: https://doi.org/10.64898/2026.01.30.702842; this version posted February 2, 2026. The copyright holder for this preprint (which was not certified by peer review) is the author/funder, who has granted bioRxiv a license to display the preprint in perpetuity. It is made available under aCC-BY 4.0 International license.

There are two redundancies in our algorithm which contribute to stabilizing performance in the no local connectivity model. First, one such redundancy is using the human subjective map, which contains spatially smooth segments, as the initial guess. This ensures that $\gamma_{i,k}^{(0)}$ in equation S43 is already smoothed, which influences the $q_{i,k}^{(t)}$ at later iterations in equation S45. Indeed, when we considered a no local connectivity model with random initialization, it did not converge to meaningful segmentation maps. This further corroborates that the initialization to a smooth map contributes to obtaining a smooth map at convergence.

The second redundancy is the fact that the model does not output the reaction time or choice for a single pair of pixels. We average the model reaction time and response at pair i, j over multiple pseudopairs, namely pairs of coordinates that are close to i and j (see Methods). Averaging over pseudopairs introduces some spatial smoothing, reducing the difference between the full IBI model and the no local connectivity model. Nonetheless, we can use these pseudopairs to compare the two models in greater depth. We introduce a new quantity we call spatial confidence, which quantifies the agreement across pseudopairs for a certain choice. For example, if 51% of pseudopairs around pixels x and y yield positive evidence this would lead the model to output a "same segment" response for pixels x,y. If, on the other hand, 99% of pseudopairs around pixels w and z have positive evidence there is clearly more spatial confidence for the w, z pair than the x, y pair, but the model outputs the same binary "same segment" response. As expected from the lack of spatial prior in the no local connectivity model, we found that its spatial confidence was lower than the IBI model(Table 2).

	IBI	no local connectivity
same segment	0.87	0.79
different segments	0.95	0.89

Table 2: Comparing spatial confidence for the IBI model and the no local connectivity model. Left-column values are higher than right-column values in both rows. The extent to which these number are still quite similar is due to the initial guess, so it stands to reason that the local-connectivity prior improves on spatial confidence.

This choice to have the majority of pseudopairs yield a response stabilizes the no local connectivity model in a way that would be perturbed if we were to, say, consider only the response of the central pseudopair. The full IBI model is not susceptible to this perturbation (Supp. Fig. 14a).

To further elucidate the difference between IBI and the no local connectivity model, we performed a temporal PCA where the evidence trace for each pseudopair was treated as an observation and each time-step was treated as a feature to find temporal basis functions for each model. We found that the no local connectivity model's traces displayed one order of magnitude more variance around the first-component basis function compared to the full IBI model. We also found that IBI's first-component basis function was more complex and showed oscillatory behavior (Supp. Fig. 14b).

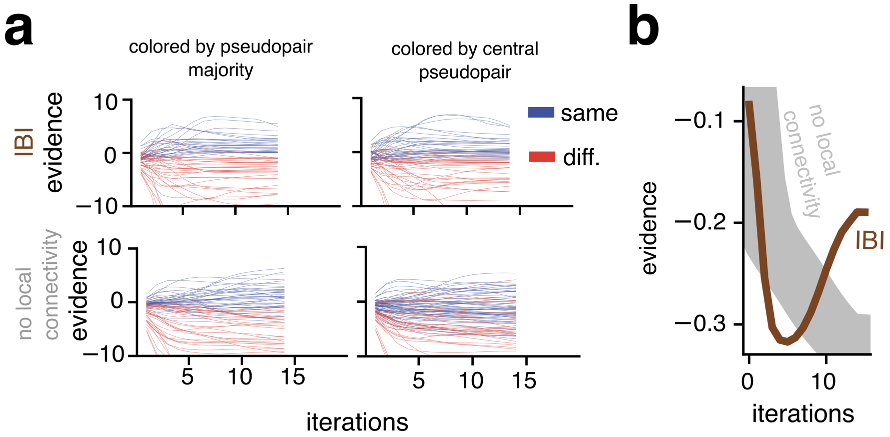

Supplementary Figure 14: The no local connectivity model is not robust to small spatial perturbations. a, Top row: evidence traces for the full IBI model colored by the pseudopair majority (left), versus the central pseudopair (right). The two partitions are almost equivalent. Bottom row: evidence traces for the no local connectivity model colored by the pseudopair majority (left) versus the central pseudopair (right). The partition coloring by the central pseudopair shows many same segment traces yielding negative evidence, because evidence from surround pixels is not in accordance with center pixels. **b**, EM iterations (time) are plotted on the x-axis, while evidence is plotted on the y-axis. Note that the amplitude of the evidence is arbitrary here as the actual evidence is a scalar multiple of these plotted basis functions. The thickness of each plotted line indicates the variance of evidence traces around the basis function. In the no local connectivity model, there is 10 times more variance than in the IBI model.

Ultimately these differences may be obscured by averaging over pseudopairs to predict behavioral proxies such as reaction time, but more work could be done to develop testable hypothesis about spatial confidence and variability and determine which model is better aligned to the empirical data.

bioRxiv preprint doi: https://doi.org/10.64898/2026.01.30.702842; this version posted February 2, 2026. The copyright holder for this preprint (which was not certified by peer review) is the author/funder, who has granted bioRxiv a license to display the preprint in perpetuity. It is made available under aCC-BY 4.0 International license.

E.2. No mental map model. For the random mental map we sampled the initial guess from a multinomial distribution where samples had equal probability of segment label up to the prescribed number of segments K:

$$c_{i,k}^{(t=0)} \sim \mathrm{Mult}[(1/K, ..., 1/K)_K] \tag{S46}$$

$$\pi_{i,k}^{(t=0)} = \begin{cases} 1-\epsilon & \text{if} \quad k = c_{i,k} \\ 0+\epsilon/(K-1) & \text{if} \quad k \neq c_{i,k} \end{cases} \tag{S47}$$

F. Model convergence. Having defined the initial state of our algorithm (the "mental map"), and the processing at each time step we now define model convergence.

F.1. The iterative timescale. As parameters are updated at each time step, $\boldsymbol{\pi}^{(t)}$ and $\boldsymbol{\theta}^{(t)}$ can be used to compute the value of the observation log-likelihood per time–step $\log p\left(\mathbf{X} \middle| \mathbf{\Pi}^{(t)}, \boldsymbol{\theta}^{(t)}\right)$ shortened $\mathcal{L}^{(t)}$. We define the time of convergence, t^*

$$t^* : \mathcal{L}^{(t)} - \mathcal{L}^{(t-1)} < \delta\mathcal{L} \tag{S48}$$

Where $\delta\mathcal{L}$ is a small, positive constant.

Therefore, we notate the per-pixel segment probabilities at convergence as $\pi_{i,k}^{(t^*)}$. Conceptually, we can think of t^* as the time at which further iteration does not meaningfully increase observation likelihood. In other words, after time t^* there is no informational benefit to the computational cost of iteration.

G. Evidence for "same segment". Thus far, we have described FlexMM which provides us with $\pi_{i,k}^{(t)}$, *i.e.* the segment probabilities for each pixel, up until convergence at $\pi_{i,k}^{(t^*)}$ to get from these to evidence about the "same segment" hypothesis we rewrite equations 3–4 from the main text, Methods.

$$\pi_{ij}^{(t)} = \sum_{k}^{K} \pi_{i,k}^{(t)} \pi_{j,k}^{(t)} \tag{S49}$$

$$\boldsymbol{E}_{ij}(t) = \left[\log\left(\pi_{ij}^{(t)}\right) - \log\left(1 - \pi_{ij}^{(t)}\right)\right] \tag{S50}$$

π_{ij} in equation S49 is the probability that two pixels i and j belong to the same segment. From this probability we use the log-likelihood ratio as evidence, notated $\boldsymbol{E}_{ij}$. Note that although $\boldsymbol{E}$ is not a vector quantity, we use boldface to emphasize that $\boldsymbol{E}$ is generated by the IBI model using multi-dimensional inference procedures. The use of the log-likelihood ratio is a principled choice in models of decision–making, including the DDM, as will be further discussed in the upcoming Section 12.

bioRxiv preprint doi: https://doi.org/10.64898/2026.01.30.702842; this version posted February 2, 2026. The copyright holder for this preprint (which was not certified by peer review) is the author/funder, who has granted bioRxiv a license to display the preprint in perpetuity. It is made available under aCC-BY 4.0 International license.

Supplementary Note 12: Drift Diffusion modeling

Previous work in decision modeling states that an optimal observer—that is one who responds the fastest given an acceptable maximum error rate—can be modeled using the drift diffusion model (DDM)[29]. The goal of a DDM is to predict reaction time distributions per choice–type[30]. In this work, we do fit DDMs to reaction time distributions, but our goal is not prediction. Instead we wish to compare the widely-adopted stochastic linear dynamics of a DDM to the nonlinear dynamics of IBI. For fair comparison, we developed an image-computable DDM and here we review the assumptions we followed in order to do so.

A. Sequential log-probability ratio test. First, we define the fundamental assumptions of DDMs. DDMs model decision-making as a sequential analysis problem, in which evidence, ξ, about the stimulus is accumulated over s sequential samples (*i.e.* $\xi_0, \xi_1, ..., \xi_s$). The optimal way to consider this evidence is using the sequential probability ratio test[29,31–33], where h_y is the hypothesis that two cues are in the same segment while h_n is the hypothesis that two cues are in a different segment:

$$L_s = \log\left(\frac{p\left(\xi_{0\to s} \mid h_\mathrm{y}\right)}{p\left(\xi_{0\to s} \mid h_\mathrm{n}\right)}\right) \tag{S51}$$

Assuming that samples are independent we can take the limit approaching a differential amount of time (dt):

$$\lim_{s\to dt}\left(L_s\right) \equiv \ell = \log\left(\frac{p\left(\xi_{dt} \mid h_\mathrm{y}\right)}{p\left(\xi_{dt} \mid h_\mathrm{n}\right)}\right) \tag{S52}$$

In DDMs, a simplifying assumption is made that because evidence is drawn from a large population of neurons perturbed by noise, the central limit theorem applies, and the differential log–likelihood can be re-parametrized as a Gaussian with mean m and variance η^2 (see Bogacz[29] Appendix A for a more detailed derivation).

$$\ell = m + \eta dW_t \tag{S53}$$

Classical DDMs use a stochastic process called the Wiener process to model noise. The Wiener process $W(t)$ is introduced such that $dW \sim \sqrt{dt}\cdot\mathcal{N}$ where $\mathcal{N}$ is a Gaussian with zero mean and unit variance. Following from the independent samples assumption made earlier: for $0 < s < t < u < v$, $W(v) - W(u)$ is independent from $W(t) - W(s)$[29]. Using the Wiener process we can reparametrize log-likelihood ratio L as a function of time $L(t)$:

$$L(t) = z + m\int_0^t ds + \eta\int_0^t dW_s \tag{S54}$$

Where z is introduced as a starting point bias. Integral notation makes clear that evidence accumulation (a.k.a. evidence integration) refers to averaging out noise over time, but here on we write $L(t)$ without integrals for simplicity, like so:

$$L(t) = z + mt + \eta W(t) \tag{S55}$$

B. Iterative and Integrative timescales. Treatment of the differential log–likelihood ℓ is where FlexMM differs from classical DDMs. In FlexMM, the dynamics of ℓ is determined by the information that FlexMM extracts from the image, and thus ℓ is affected by the connectivity of the graph. Comparing $L(t)$ to $\boldsymbol{E}_{ij}(t)$ from equation S50 in Supplementary Note 11, we can appreciate that they are both log-probability ratios. Thanks to this shared probabilistic formulation, we were able to further develop bespoke DDMs that leverage image statistics while eliminating the nonlinear within-trial dynamics of iteration in IBI.

The intuitive approach was to assume that we can substitute the parameters in $L(t)$ with value of the *unchanging* log-likelihood at iteration convergence $\boldsymbol{E}_{ij}(t^*)$, divided by t^*. We introduce a new variable for differential evidence in the integrative timescale:

$$\mathcal{E}_{ij} = \frac{\boldsymbol{E}_{ij}^{(t^*)}}{t^*} \tag{S56}$$

This quotient is the average value of sensory evidence during the iterative timescale. Notionally, we divide by t^* to keep our units consistent with m. Practically, this division ensures that stimuli that take longer to converge do not have their evidence count for more, which would be counter–intuitive.

bioRxiv preprint doi: https://doi.org/10.64898/2026.01.30.702842; this version posted February 2, 2026. The copyright holder for this preprint (which was not certified by peer review) is the author/funder, who has granted bioRxiv a license to display the preprint in perpetuity. It is made available under aCC-BY 4.0 International license.

Therefore, technically speaking, $L(t)$ in only defined for $t > t^*$. Following the assumptions of sequential analysis, this creates a decision-making model in which the integrative timescale follows the iterative timescale. The model which yields a better fit is deemed to "set the tempo". In other words, it is a better model of the slow step of the decision-making process. That is, if DDM is a better model, extracting the evidence from the input image either takes the same amount of time for all pairs or is much faster (and thus has less influence on reaction times) than the process of averaging out noise. Conversely, if IBI is a better model, reaction times are largely determined by the dynamic process that extracts evidence from the sensory input, whereas additional noise is negligible. See Table 3 for a side–by–side comparison of timescales.

Note that neither $\boldsymbol{E}_{ij}(t)$ nor $L_{ij}(t)$ are closed-form continuous expressions. However, both the models can be thought of as using a first-order numerical integration procedure to approximate a continuous evidence function.

IBI (for $t \to t^*$)	DDMs (for $t^* \to T$)
$\boldsymbol{E}_{ij}(t) = \left[\log\left(\pi_{ij}^{(t)}\right) - \log\left(1-\pi_{ij}^{(t)}\right)\right]$	$L_{ij}(t) = z + \alpha \cdot m_{ij} t + \eta W(t)$
Decision rules:	Decision rules:
$\rho_1 = \{0 \le t \le t^* : \lvert \boldsymbol{E}_{ij}(t)\rvert \ge b\}$ $\rho_2 = \left\{0 \le t \le t^* : \left\lvert \frac{\partial}{\partial t}(\boldsymbol{E}_{ij})\right\rvert \le a \lvert \boldsymbol{E}_{ij}\rvert \right\}$ **(S57)**	$\rho_3 = \{t^* \le t \le T : \lvert L_{ij}(t)\rvert \ge \beta\}$
Fittable Parameters (ψ):	Fittable Parameters (ψ):
$\psi = (a, b)$	$\psi = (\alpha, \beta)$
Decision time distributions:	Decision time distributions:
$\hat{\mathbf{t}}_{d,\mathrm{y}} = \inf\{(\rho_1 \cup \rho_2) \cap (\boldsymbol{E}_{ij}(t) > 0)\}$ **(S58)** $\hat{\mathbf{t}}_{d,\mathrm{n}} = \inf\{(\rho_1 \cup \rho_2) \cap (\boldsymbol{E}_{ij}(t) < 0)\}$ **(S59)**	$\hat{\mathbf{t}}_{d,\mathrm{y}} = \inf\{(\rho_3) \cap (L_{ij}(t) > 0)\}$ **(S60)** $\hat{\mathbf{t}}_{d,\mathrm{n}} = \inf\{(\rho_3) \cap (L_{ij}(t) < 0)\}$ **(S61)**

Table 3: A side-by-side comparison of the iterative and integrative timescales

C. Types of DDMs. All the DDMs we use can be defined with the following parameterization, where $W_{ij}(t)$ indicates that each unique pair (i, j) is an independent draw from the Wiener process:

$$L_{ij} = z_{ij} + \alpha \cdot m_{ij} + \eta W_{ij}(t) \tag{S62}$$

See Table 4 for how we modify substitutions of $\mathcal{E}_{ij}$ to create the different DDMs reported in our main text as well as in Supplementary Note 6.

bioRxiv preprint doi: https://doi.org/10.64898/2026.01.30.702842; this version posted February 2, 2026. The copyright holder for this preprint (which was not certified by peer review) is the author/funder, who has granted bioRxiv a license to display the preprint in perpetuity. It is made available under aCC-BY 4.0 International license.

z_{ij}	m_{ij}	Heuristic	Nomenclature in main text
0	1	α is decoded from data	Base DDM
0	$\langle \mathcal{E}_{ij} \rangle_{\hat{\mathrm{y}^*}/\hat{\mathrm{n}^*}}$	weight by choice-type	choice–weighted DDM
$\mathcal{E}_{ij}$	$\mathcal{E}_{ij}$	weight per trial	image–computable DDM/trial–weighted DDM
0	$\mathcal{E}_{ij}$	weight drift rate per trial	reduced trial–weighted DDM
$\mathcal{E}_{ij}$	$\langle \mathcal{E}_{ij} \rangle_{\hat{\mathrm{y}^*}/\hat{\mathrm{n}^*}}$	weight starting point per trial	reduced trial–weighted DDM

Table 4: Types of DDMs. The angle–bracket notation indicates an average. The notation $\hat{\mathrm{y}^*}/\hat{\mathrm{n}^*}$ indicates that evidence is averaged per the choice type of the model, at convergence; y: same segment, n: different segments.

bioRxiv preprint doi: https://doi.org/10.64898/2026.01.30.702842; this version posted February 2, 2026. The copyright holder for this preprint (which was not certified by peer review) is the author/funder, who has granted bioRxiv a license to display the preprint in perpetuity. It is made available under aCC-BY 4.0 International license.

Supplementary Note 13: Parameter fitting

In order for the model to generate predictions $\hat{\mathbf{t}}_d$ that are most aligned with empirical observations $\mathbf{t}_d$, we implemented a parameter fitting procedure. Only two parameters, $\psi = a, b$ for IBI or $\psi = \alpha, \beta$ for DDM, were used for the fitting while the noise η remained fixed.

To be able to compare the model's output of reaction time (which is based on an iteration index) with the continuous reaction time (based on physical time) from humans performing the task, we apply the following transformation to both model and human reaction times: $\mathbf{t}'_d = \texttt{resc}(\log(\mathbf{t}_d + 1))$. Where the $\texttt{resc}(\mathbf{x})$ function is simply $(\mathbf{x} - \mathbf{x}_{\min})/(\mathbf{x}_{\max} - \mathbf{x}_{\min})$.

The goal is to maximize the likelihood that a human decision time $\mathbf{t'}_d$ comes from the distribution of model reaction times $\hat{\mathbf{t}}'_d$. State-of-the-art DDMs use an analytical solution to construct the PDF and compute a loss $\hat{f}\left(\hat{\mathbf{t}}'_d|\psi\right)$ [34,35]. While this would be possible for the base DDM model, it is not applicable to all models so we opted to empirically construct CDFs for loss instead: $\hat{F}\left(\hat{\mathbf{t}}'_d|\psi\right)$. We can use the following empirical likelihood function to calculate the likelihood that a distribution of human reaction times $\mathbf{t}'_d$ comes from the model CDF $\hat{F}$ [36]:

$$p(\mathbf{t}'_d|\hat{F},\psi) = \prod_{n=1}^{N_T} \frac{\hat{F}\left(t'_{d,n}|\psi\right) - \hat{F}\left(t'_{d,n} - \delta\left(t'_{d,n}\right)|\psi\right)}{\delta\left(t'_{d,n}\right)} \tag{S63}$$

where the product is over all trials, and $\delta(t'_{d,n})$ is the time difference with the closest observation smaller than $t'_{d,n}$. For numerical stability, we optimize the negative log–likelihood $-\mathscr{L}\left(\mathbf{t}'_d, \hat{F}, \psi\right)$.

$$-\mathscr{L}\left(\mathbf{t}'_d, \hat{F}, \psi\right) = \sum_{n=1}^{N_T} \log\left[\hat{F}\left(t'_{d,n}|\psi\right) - \hat{F}\left(t'_{d,n} - \delta\left(t'_{d,n}\right)\big|\psi\right)\right] - \log\left(\delta\left(t'_{d,n}\right)\right) \tag{S64}$$

The second term is not a function of the parameters so we ignore it. For the final loss function, we apply the above equation independently to each set of choices

$$-\mathscr{L}_{\text{total}} = -\mathscr{L}\left(\mathbf{t}'_{d,\text{y}}, \hat{F}_{\text{y}}, \psi\right) - \mathscr{L}\left(\mathbf{t}'_{d,\text{n}}, \hat{F}_{\text{n}}, \psi\right) \tag{S65}$$

Note that the subscripts y and n indicate the "same segment" subset, and the "different segments" subset as defined by human choices, ignoring the choices of the model. This is because the choices made by the model at specific trials were not expected to match with the human's, the objective was simply to match reaction time distributions per choice type. Equation S65 is not differentiable with respect to the parameters. Therefore we used Markov-Chain Monte-Carlo (MCMC) global minimization methods [37,38]. Starting from an initial guess (e.g., for IBI, $\psi^{(0)} = a^{(0)}, b^{(0)}$) we compute the negative log-likelihood $-\mathscr{L}_{\text{total}}$, which is then iteratively minimized. We heuristically set the following bounds for the MCMC optimizer on parameters in ψ: $a \in (0,1]$, $b \in (0,5]$, $\alpha \in (0,50])$, $\beta \in (0,5]$. We also found that the minimization of loss improved when multiple initial guesses of ψ were used. We used two different initial guesses.

By computing this minimization we are finding parameters that maximize the likelihood of human decision times being predicted by our model. For fair comparison across models we cross-validated each model fit with a 5-fold cross validation per case.

bioRxiv preprint doi: https://doi.org/10.64898/2026.01.30.702842; this version posted February 2, 2026. The copyright holder for this preprint (which was not certified by peer review) is the author/funder, who has granted bioRxiv a license to display the preprint in perpetuity. It is made available under aCC-BY 4.0 International license.

Supplementary Note 14: IBI model extensions

The choices made in our modeling (e.g. using a convolutional neural network for feature extraction or learning parametric distributions) are rooted in existing literature, and allow for controlled model comparison with alternatives that shed light on exactly what aspects of the model are useful for capturing the spatiotemporal dynamics of human segmentation. Our model lays the foundation however, for a number of extensions which could potentially improve the quantitative performance of IBI to predict reaction times, detailed below.

Extending feature extraction. We use the deep convolutional neural network VGG-19 for feature extraction because it has a receptive field like architecture that mimics early visual cortex[3,4]. In computer vision however, the field has moved away from deep convolutional neural networks and towards more powerful transformer models from which segmentation features which include semantic information may emerge[39]. These segmentation features can easily be plugged into our iterative generative model which could then predict reaction times using iteration index.

Extending decision rules and parameters to fit reaction time data. We used the simplest possible decision rules given our goal of comparing to DDMs, which led our IBI model to have only two fittable parameters to fit reaction times. Given there are hundreds of trials, we could add decision rules and new parameters. One example of a new decision rule would be to take into account the trial number, *i.e.* the sequential order in which pairs of cues are presented during the experiment (see Supp. Fig. 15).

Finally, consistent with the DDM literature, our objective function is minimized to match the overall reaction time distribution rather than predicting reaction times per trial. Instead of matching empirical CDFs, we could use a more standard loss function, for example the squared error.

bioRxiv preprint doi: https://doi.org/10.64898/2026.01.30.702842; this version posted February 2, 2026. The copyright holder for this preprint (which was not certified by peer review) is the author/funder, who has granted bioRxiv a license to display the preprint in perpetuity. It is made available under aCC-BY 4.0 International license.

Supplementary Note 15: Reaction times decrease throughout the experimental block

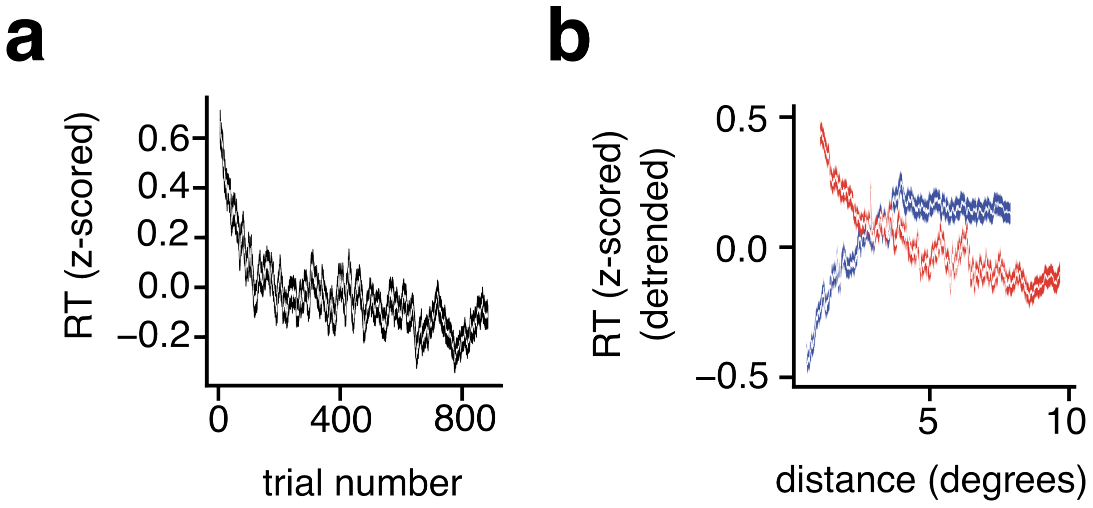

Supplementary Figure 15: **a**, z-scored reaction time vs. ordered trial number. Trials are aggregated across all cases. The white line represents the mean within a sliding window of 600 trials. Black shaded area: s.e.m. **b**, Same as Figure 2a, bottom, with detrended data (*i.e.* removing the mean trend of panel **a**; details in the text below).

We observe that RTs decrease as the task goes on (Supp. Fig. 15a). To ensure that the presence of this trend (which is unrelated to the distance between cues) did not affect the RT correlations with distance, we detrended the RT data and verified that results did not change. To detrend the data, we fit a decaying exponential to the z-scored RT vs. trial number, and then subtracted the predictions made by this exponential from the empirical z-scored RT values. Supp. Fig. 15b shows that this detrending procedure did not change our findings.

bioRxiv preprint doi: https://doi.org/10.64898/2026.01.30.702842; this version posted February 2, 2026. The copyright holder for this preprint (which was not certified by peer review) is the author/funder, who has granted bioRxiv a license to display the preprint in perpetuity. It is made available under aCC-BY 4.0 International license.

References

1. Hubert, L. & Arabie, P. Comparing partitions. *J. Classif.* **2**, 193–218 (1985).
2. Ruscio, J. Constructing confidence intervals for spearman's rank correlation with ordinal data: A simulation study comparing analytic and bootstrap methods. *J. Mod. Appl. Stat. Methods* **7**, 416–434 (2008).
3. Simonyan, K. & Zisserman, A. Very deep convolutional networks for large-scale image recognition. *3rd International Conference on Learning Representations* (2015).
4. Cadena, S. A. *et al.* Deep convolutional models improve predictions of macaque V1 responses to natural images. *PLoS Comput. Biol.* **15**, e1006897 (2019).
5. Schrimpf, M. *et al.* Integrative benchmarking to advance neurally mechanistic models of human intelligence. *Neuron* **108**, 413–423 (2020).
6. Vacher, J., Launay, C. & Coen-Cagli, R. Flexibly regularized mixture models and application to image segmentation. *Neural Netw.* **149**, 107–123 (2022).
7. Wainwright, M. J. & Simoncelli, E. Solla, S., Leen, T. & Müller, K. (eds) *Scale Mixtures of Gaussians and the Statistics of Natural Images.* (eds Solla, S., Leen, T. & Müller, K.) *Advances in Neural Information Processing Systems*, Vol. 12 (MIT Press, 1999).
8. Schwartz, O. & Simoncelli, E. P. Natural signal statistics and sensory gain control. *Nat. Neurosci.* **4**, 819–825 (2001).
9. Coen-Cagli, R., Dayan, P. & Schwartz, O. Cortical surround interactions and perceptual salience via natural scene statistics. *PLoS Comput. Biol.* **8**, e1002405 (2012).
10. Coen-Cagli, R. & Mamassian, P. Are we ready to tackle perceptual segmentation of natural scenes? *Vision Res.* **240**, 108749 (2025).
11. Festa, D., Aschner, A., Davila, A., Kohn, A. & Coen-Cagli, R. Neuronal variability reflects probabilistic inference tuned to natural image statistics. *Nat. Commun.* **12**, 3635 (2021).
12. Peel, D. & McLachlan, G. J. Robust mixture modelling using the t distribution. *Stat. Comput.* **10**, 339–348 (2000).
13. Koller, D. & Friedman, N. *Probabilistic Graphical Models: Principles and Techniques* (MIT Press, 2009).
14. Dempster, A. P., Laird, N. M. & Rubin, D. B. Maximum likelihood from incomplete data via the *em* algorithm. *J. R. Stat. Soc. Series B Stat. Methodol.* **39**, 1–22 (1977).
15. Bishop, C. M. *Pattern Recognition and Machine Learning (Information Science and Statistics)* 1 edn (Springer, 2007).
16. Eckford, A. W. The factor graph EM algorithm: applications for LDPC codes. *IEEE 6th Workshop on Signal Processing Advances in Wireless Communications, 2005* 910–914 (2005).
17. Dauwels, J., Eckford, A. W., Korl, S. & Loeliger, H. Expectation maximization as message passing - part I: principles and gaussian messages. *CoRR* **abs/0910.2832** (2009). URL http://arxiv.org/abs/0910.2832.
18. Lee, T. S. & Mumford, D. Hierarchical bayesian inference in the visual cortex. *J. Opt. Soc. Am. A Opt. Image Sci. Vis.* **20**, 1434–1448 (2003).
19. Beck, J. M., Latham, P. E. & Pouget, A. Marginalization in neural circuits with divisive normalization. *J. Neurosci.* **31**, 15310–15319 (2011).
20. Raju, R. V., Li, Z., Linderman, S. & Pitkow, X. Inferring inference. *CoRR* **abs/2310.03186** (2023). URL https://doi.org/10.48550/arXiv.2310.03186.
21. Neal, R. M. & Hinton, G. E. in *A view of the em algorithm that justifies incremental, sparse, and other variants* (ed.Jordan, M. I.) *Learning in Graphical Models*, Vol. 89 of *NATO ASI Series* 355–368 (Springer Netherlands, 1998).
22. Roweis, S. & Ghahramani, Z. A unifying review of linear gaussian models. *Neural Comput.* **11**, 305–345 (1999).
23. Salakhutdinov, R., Roweis, S. T. & Ghahramani, Z. Optimization with EM and expectation-conjugate-gradient. *ICML'03: Proceedings of the Twentieth International Conference on International Conference on Machine Learning* 672–679 (2003).
24. Friston, K. A theory of cortical responses. *Philos. Trans. R. Soc. Lond. B Biol. Sci.* **360**, 815–836 (2005).
25. Buxó, C. E. R. & Savin, C. Ranzato, M., Beygelzimer, A., Dauphin, Y. N., Liang, P. & Vaughan, J. W. (eds) *A sampling-based circuit for optimal decision making.* (eds Ranzato, M., Beygelzimer, A., Dauphin, Y. N., Liang, P. & Vaughan, J. W.) *Advances in Neural Information Processing Systems*, 14163–14175 (2021).
26. Chen, S., Jiang, L., Rao, R. P. N. & Shea-Brown, E. Oh, A. *et al.* (eds) *Expressive probabilistic sampling in recurrent neural networks.* (eds Oh, A. *et al.*) *Advances in Neural Information Processing Systems* (2023).
27. Zhang, W.-H., Wu, S., Josić, K. & Doiron, B. Sampling-based bayesian inference in recurrent circuits of stochastic spiking neurons. *Nat. Commun.* **14**, 7074 (2023).

bioRxiv preprint doi: https://doi.org/10.64898/2026.01.30.702842; this version posted February 2, 2026. The copyright holder for this preprint (which was not certified by peer review) is the author/funder, who has granted bioRxiv a license to display the preprint in perpetuity. It is made available under aCC-BY 4.0 International license.

28. Jordan, M. I. & Xu, L. Convergence results for the EM approach to mixtures of experts architectures. *Neural Netw.* **8**, 1409–1431 (1995).
29. Bogacz, R., Brown, E., Moehlis, J., Holmes, P. & Cohen, J. D. The physics of optimal decision making: a formal analysis of models of performance in two-alternative forced-choice tasks. *Psychol. Rev.* **113**, 700–765 (2006).
30. Fudenberg, D., Newey, W., Strack, P. & Strzalecki, T. Testing the drift-diffusion model. *Proc. Natl. Acad. Sci. U. S. A.* **117**, 33141–33148 (2020).
31. Neyman, J. & Pearson, E. S. IX. on the problem of the most efficient tests of statistical hypotheses. *Philos. Trans. R. Soc. Lond.* **231**, 289–337 (1933).
32. Wald, A. *Sequential analysis* (John Wiley, 1947).
33. Wald, A. & Wolfowitz, J. Optimum character of the sequential probability ratio test. *Ann. Math. Stat.* **19**, 326–339 (1948).
34. Feller, W. *An Introduction to Probability Theory and Its Applications* Vol. 1 (Wiley, 1968).
35. Navarro, D. J. & Fuss, I. G. Fast and accurate calculations for first-passage times in wiener diffusion models. *J. Math. Psychol.* **53**, 222–230 (2009).
36. Owen, A. *Empirical Likelihood* (CRC Press, 2001).
37. Wales, D. J. & Doye, J. P. K. Global optimization by basin-hopping and the lowest energy structures of Lennard-Jones clusters containing up to 110 atoms. *J. Phys. Chem. A* **101**, 5111–5116 (1997).
38. Xiang, Y., Sun, D. Y., Fan, W. & Gong, X. G. Generalized simulated annealing algorithm and its application to the thomson model. *Phys. Lett. A* **233**, 216–220 (1997).
39. Oquab, M. *et al.* Dinov2: Learning robust visual features without supervision. *Trans. Mach. Learn. Res.* **2024** (2024).
